# A Computational Recognition Analysis of Promising Prognostic Biomarkers in Breast, Colon and Lung Cancer Patients

**DOI:** 10.3390/ijms26031017

**Published:** 2025-01-25

**Authors:** Tala Bakheet, Nada Al-Mutairi, Mosaab Doubi, Wijdan Al-Ahmadi, Khaled Alhosaini, Fahad Al-Zoghaibi

**Affiliations:** 1 Molecular BioMedicine Program, King Faisal Specialist Hospital & Research Centre, Riyadh 11211, Saudi Arabia; tala@kfshrc.edu.sa (T.B.); nalmutairi28@kfshrc.edu.sa (N.A.-M.); mdoubi02@kfshrc.edu.sa (M.D.); walahmadi@kfshrc.edu.sa (W.A.-A.); 2 Department of Pharmacology & Toxicology, College of Pharmacy, King Saud University, Riyadh 11451, Saudi Arabia; kalhosaini@ksu.edu.sa; 3 Department of Molecular and Cell Biology, University of Leicester, Leicester LE1 7RH, UK

**Keywords:** prognostic biomarkers, RFS, PF, HR, breast cancer, colon cancer, lung cancer, prognostic predictors

## Abstract

Breast, colon, and lung carcinomas are classified as aggressive tumors with poor relapse-free survival (RFS), progression-free survival (PF), and poor hazard ratios (HRs) despite extensive therapy. Therefore, it is essential to identify a gene expression signature that correlates with RFS/PF and HR status in order to predict treatment efficiency. RNA-binding proteins (RBPs) play critical roles in RNA metabolism, including RNA transcription, maturation, and post-translational regulation. However, their involvement in cancer is not yet fully understood. In this study, we used computational bioinformatics to classify the functions and correlations of RBPs in solid cancers. We aimed to identify molecular biomarkers that could help predict disease prognosis and improve the therapeutic efficiency in treated patients. Intersection analysis summarized more than 1659 RBPs across three recently updated RNA databases. Bioinformatics analysis showed that 58 RBPs were common in breast, colon, and lung cancers, with HR values < 1 and >1 and a significant Q-value < 0.0001. RBP gene clusters were identified based on RFS/PF, HR, *p*-value, and fold induction. To define union RBPs, common genes were subjected to hierarchical clustering and were classified into two groups. Poor survival was associated with high genes expression, including *CDKN2A, MEX3A, RPL39L, VARS, GSPT1, SNRPE, SSR1*, and *TIA1* in breast and colon cancer but not with lung cancer; and poor survival was associated with low genes expression, including *PPARGC1B, EIF4E3,* and *SMAD9* in breast, colon, and lung cancer. This study highlights the significant contribution of *PPARGC1B*, *EIF4E3*, and *SMAD9* out of 11 RBP genes as prognostic predictors in patients with breast, colon, and lung cancers and their potential application in personalized therapy.

## 1. Introduction

Cancer is one of the deadliest illnesses in the world. According to a World Health Organization (WHO) report in 2020, 10 million new cancers are diagnosed globally each year, and this number is expected to increase to 20 million over the next 17 years. The most common life-threatening tumors are breast, colorectal, lung, prostate, and stomach tumors, which do not respond well to treatment.

Breast, colon, and lung carcinomas are classified as aggressive tumors with poor relapse-free survival (RFS), poor progression-free survival (PF), and poor hazard ratios (HRs) despite extensive therapy. A recent report on the cancer burden on member states of the European Union suggests that 4 million new cancer cases (excluding non-melanoma skin cancer) and 1.9 million cancer-related casualties occur annually [1]. The most common causes of cancer deaths are lung (0.38 million), colorectal (0.25 million), and breast (0.14 million).

Consequently, experts have always argued that research, information, and awareness are crucial for cancer prevention, early detection, and strategic treatment options. Global gene analyses have confirmed an association between genes, diseases, and drugs [2]. Therefore, it is essential to identify a gene expression signature that correlates with RFS, PF, and HR in order to predict treatment efficiency.

Precision medicine is used to assess the epigenetic regulation of disease at the molecular level in an individual patient, and this helps researchers to tailor appropriate and optimal therapies that can be used in addition to current therapies or as monotherapy based on each patient’s unique omics features, maximizing drug efficacy, and minimizing adverse drug reactions. However, the fragmentation and heterogeneity of the available data make it difficult to obtain first-hand information.

Regulation of gene expression is mostly performed by RNA-binding proteins (RBPs), which bind to unique RNA-binding sites and alter the fate or function of bound RNAs. Over the years, several hundred RBPs have been identified and studied for their critical roles in regulating transcriptional and post-transcriptional gene expression, and their unique involvement in cellular processes. RBPs contribute to RNA processing in major human diseases, including neurodegenerative diseases, cancer and muscular atrophies [3,4,5,6,7]. However, their involvement in cancer is not yet fully understood.

In this study, we used computational bioinformatics to classify the correlation between the expression level, survival, and HR risk factors of RBPs in solid cancers. We aimed to identify molecular biomarkers that could help predict disease prognosis and improve the therapeutic efficacy in patients.

A total of 1659 RBP gene summaries were obtained from three different RNA databases: RBPome (1344 genes), Census (1542 genes), and RBPDB (416 genes) [8,9]. A total of 58 common RBP gene signatures were collected across breast, colon, and lung cancers. Union RBP gene signatures of 11 genes were defined by exposing the common (58) genes to hierarchical clustering with RFS, PF, HR, *p*-value, and fold of induction. Based on the clustering results, four clusters were identified. In these clusters, RBPs were classified as having poor survival with high-risk HR genes (*CDKN2A*, *MEX3A, RPL39L,* and *VARS*) or poor survival with low-risk HR genes (*GSPT1, SNRPE, SSR1, TIA1, PPARGC1B, EIF4E3,* and *SMAD9*).

This study highlights the significant contribution of 11 RBP genes as prognostic predictors in patients with breast, colon, and lung cancers and their potential application in personalized therapy. Here, we present a correlation between the upregulation and downregulation of RBPs in cancer development. In summary, poor survival is associated with *PPARGC1B, EIF4E3*, and *SMAD9* low gene expression in breast, colon and lung cancer.

## 2. Results

### 2.1. Union RBP Intersection Master List and Gene Clustering

**Figure 1:**
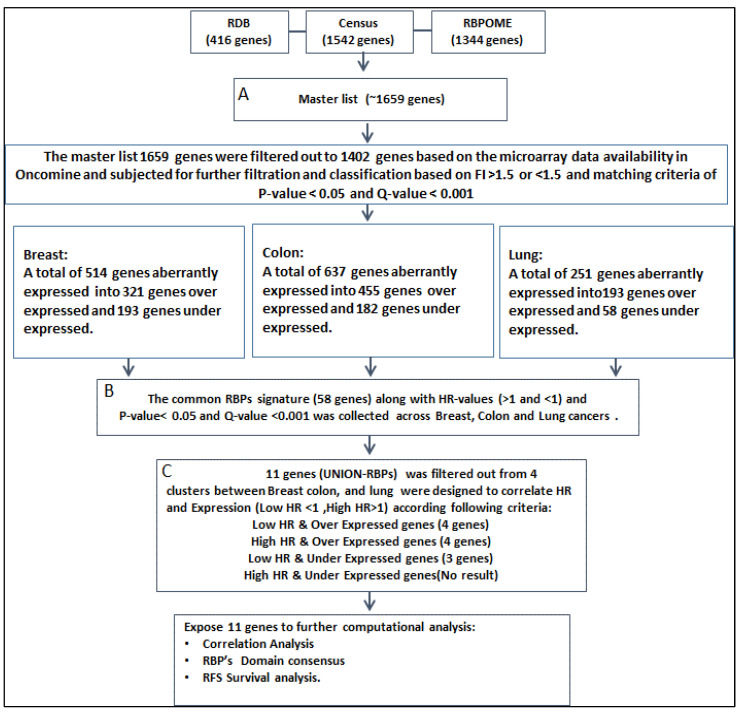
Study layout shows steps of the union RBPs list compilation. (**A**) Master list of 1659 genes was intersected out of 3302 compiled genes from three databases including RBPome, Census and RBPDB. (**B**) In total, 58 common RBP gene signatures out of 1659 genes were segregated across breast, colon and lung cancers and exposed to further filtration along with HR values (>1 and <1) and *p*-value < 0.05 and Q-value < 0.001. (**C**) In total, 11 union RBP gene signatures were filtered out of 4 hierarchical clusters.

A total of 3302 genes from three RBP databases were combined to form the master list, which comprised 1659 unique genes identified across breast, colon, and lung cancers. The layout of the analysis is shown in (Figure 1). The intersection between these three databases was conducted to give a set of 376 genes (Supplemental Table S4). However, the study emphasized the combined master list including 376 intersected genes.

The master list of 1659 genes was filtered out to 1402 genes based on the microarray data availability in Oncomine (www.oncomine.com). The downloaded RBP master list was subjected to further filtration and classification based on fold induction: >1.5 and <−1.5 and matching criteria of *p*-value < 0.05 and Q-value < 0.001 (Table 1). The updated master list of RBPs, which comprised 1402 genes, was defined. Approximately 321 of the 514 breast cancer genes were classified as upregulated, and 193 were classified as downregulated. For colon cancer, 455 of the 637 genes were classified as upregulated and 182 were classified as downregulated. Of 251 lung cancer genes, 193 were classified as upregulated and 58 were classified as downregulated.

To define the 58 common RBP signatures, the updated 1402 genes from the master list were subjected to the clinical data analysis that includes HR values, *p*-values and Q-values for each cancer type from the work of Balazs Gyorffy (Supplemental Table S5). Our criteria were based on the value of fold induction where >1.5 is considered as overexpressed and <−1.5 is considered as underexpressed. This was followed by filtration of HR values greater than 1 and less than 1, with significant +*p*-values of <0.05 and Q-value < 0.001.

**Figure 2:**
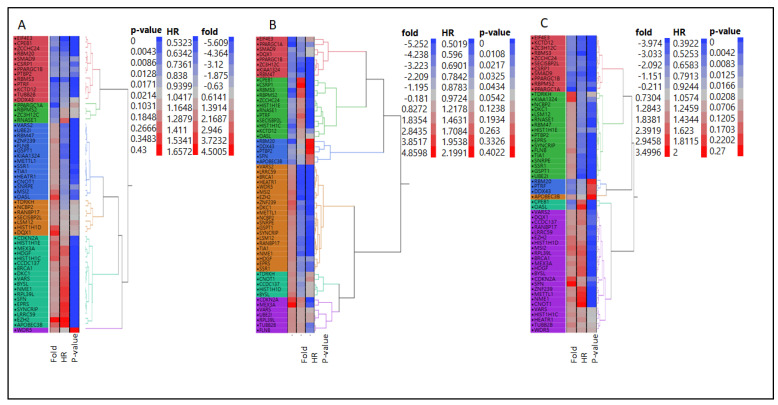
Hierarchical clustering heat map graphs. The common RBPs signature (58 genes) expression values, HR and *p*-values were subjected to be clustered into 6 clusters for (**A**) breast cancer, (**B**) colon and (**C**) lung cancer.

Supervised hierarchical clustering visualization was performed using JMP^®^ (Version 12, SAS Institute Inc., Cary, NC, USA, 1989–2019) for union RBP genes (Figure 2 and Table 2). The inputs to the model were up/downregulated RNA data for each cancer type. Specifically, the fold change of tumor to normal tissues (T/N), HR, and *p*-value for each RBP served as the input for the clustering.

Representative HR values and *p*-values were aligned in the microarray gene expression data of the common RBP gene list (see Supplemental Table S1). For each cancer, a set of 58 gene expression values, HR, and *p*-values were subjected to hierarchical clustering into six clusters (Figure 2A–C). Six clusters were identified for breast, colon, and lung cancers (Table 2A–C). After clustering each cancer, genes were grouped in clusters of either good/poor survival or up- or downregulated genes.

As this study aimed to identify key prognostic predictor genes associated with survival conditions, HR status, and gene expression, the first filtration was based on HR values followed by gene expression. Four classes were designed to categorize these genes based on their HR values and gene expression (Table 3). Class 1 (highest HR values and upregulated genes [HR > 1 and FI > 1.5]), Class 2 (lowest HR values and upregulated genes [HR < 1 and FI > 1.5]), Class 3 (highest HR values and downregulated genes [HR > 1 and FI < −1.5]), and Class 4 (lowest HR values and downregulated genes (HR < 1 and FI < −1.5]).

For example, Class 1 in the breast cancer cluster represented in Cluster 5 contained 18 genes with average FI and HR values of 2.2835 and 1.4626, respectively, and a *p*-value < 0.001. Similarly, in colon cancer, cluster 6 contained seven genes with average FI and HR values of 22.4175 and 1.3280, respectively, with a *p*-value ~ 0.05. In lung cancer, cluster 6 contained 24 genes with average FI and HR values of 1.9161 and 1.5380, respectively, with a *p*-value < 0.01.

In Class 1, *CDKN2A, MEX3A, RPL39L*, and *VARS* had HRs of >1 and FIs of >1.5. Class 2 also had common genes (*GSPT1, SNRPE, SSR1,* and *TIA1*) with HRs < 1 and FIs > 1.5. Class 3 had no common genes with HRs > 1 and high FIs < −1.5. Class 4 had common genes (*PPARGC1B, EIF4E3,* and *SMAD9*), with HRs < 1 and FIs < −1.5. Therefore, the following 11 genes were compiled from the four classes: *MEX3A, CDKN2A, RPL39L, VARS, GSPT1, SNRPE, SSR1, TIA1, PPARGC1B, EIF4E3,* and *SMAD9*. The mean expression levels of each gene across the three types of cancer, their HR values, and consensus targets are shown in (Table 4).

### 2.2. The Functional Analysis of the Union RBP Consensus Targets and the Suggested Protein–Protein Network Interactions:

**Figure 3:**
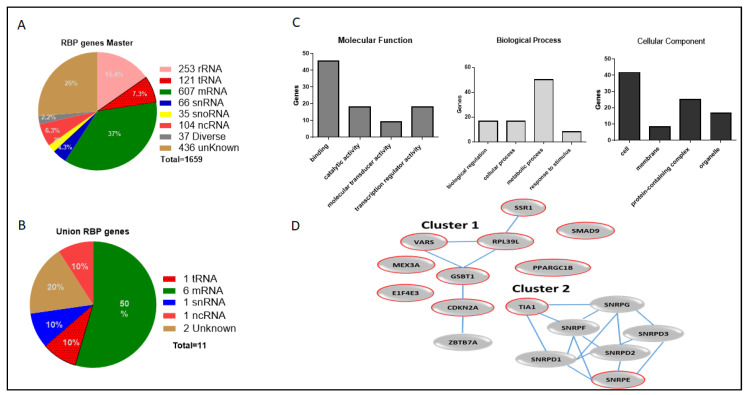
Functional analysis in human cells: (**A**) Pie chart representing the consensus targets of the RBPs master genes. (**B**) Pie chart representing the domain consensus of the union RBP genes list. (**C**) The union RBP genes’ functional classifications including molecular, biological process and cellular component. (**D**) Network representing the current protein–protein interaction of the union RBP genes, the promising prognostic biomarkers were circled with red line.

A pie chart was used to visualize the consensus targets of the master RBPs and the gene signatures of the union RBPs in human cells (Figure 3A,B). Interestingly, there were increases in the mRNA, tRNA, snRNA, and ncRNA consensus targets in the union RBPs compared to the master RBP list. However, there was a reduction in rRNA percentages and unknown consensus targets in union RBPs compared to the master RBP list. This may explain the diversity of the consensus domains. Therefore, there are no specific consensus targets for RBPs involved in cancer development or therapy rejection.

To determine the molecular and biological functions and mechanisms of the union of RBP gene signatures, we used PANTHER GO unifying biology analysis and STRING11 software.

Three functional classifications were defined for: molecular, biological, and cellular processes. The binding of indirect targets, catalytic activity, and transduction regulator activity enhanced the molecular function of the union RBPs. In the biological process, 50% of the RBPs were involved in metabolic processes and 41% were found in the cytoplasmic compartment (Figure 3C). Union genes were input into STRING11 to analyze possible interactions between the network and experimental protein–protein interactions to better visualize and understand the functions of the union RBPs. Their molecular functions have shown that they can bind to several RNAs, heterocyclic compounds, organic compounds, cyclic compounds, and translation factors. In biological processes, there is an improvement in the regulation of translation, biosynthesis of cellular nitrogen compounds and macromolecules, gene expression, and peptide metabolism. Cellular components include cytoplasmic, ribonucleic protein, and protein-containing complexes.

Moreover, the union RBPs obtained 11 PPI nodes, five edges, and a PPI enrichment *p*-value < 0.019. In addition, the STRING11 database was built on different tandem affinity purification assays, co-immunoprecipitation assays, yeast two-hybrid assays, and affinity chromatography assays that demonstrated protein–protein affinity interactions, which gave us the results of two main clusters. The first cluster consisted of five genes, *CDKN2A, GSPT1, SSR1, RPL39L*, and *VARS*. The second cluster demonstrated protein interactions between *TIA1, SNRPE*, and others. *MEX3A, SMAD9, PPARGC1B,* and *EIF4E3* were not involved in protein network interactions (Figure 3D).

### 2.3. Cross-Correlation of RBPs

The expression of 11 genes was obtained from the Oncomine database to identify potential cross-correlations between the RBP gene signatures. Multivariate similarity was calculated using SAS Institute Inc. 2013 SAS Enterprise Guide ^TM^ 6.1 across multiple genes. A volcano plot was obtained by plotting the R-values of the common 58 upregulated and downregulated RBP genes on the *x*-axis and the base 10 logarithm of their corresponding *p*-values on the *y*-axis. *p*-values < 0.00001 were considered and reported as base 10 logarithms (*p*-value of 0.00001 = 4).

**Figure 4:**
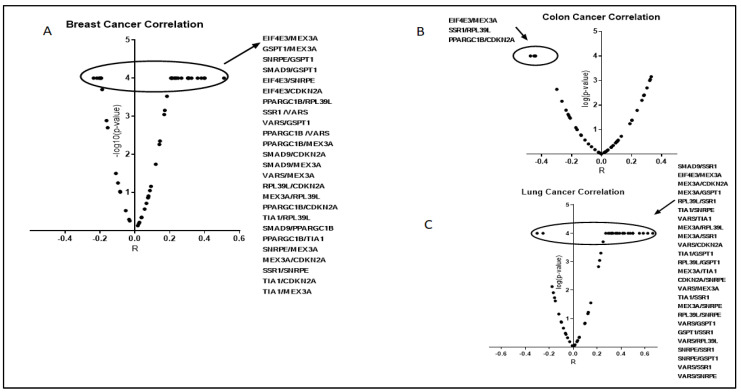
Cross correlations between the union RBP genes signature across multiple genes were determined for; (**A**) breast, (**B**) colon and (**C**) lung cancer. Volcano plot is shown by plotting the R-values on the *x*-axis and their significance *p*-values as corresponding –Log_10_ (*p*-values) on the *y*-axis.

In colon cancer, most likely because of the lack of data, cross-correlation identified only three correlating groups (*EIF4E3/MEX3A, SSR1/RPL39L*, and *PPARGC1B/CDKN2A*). The cross-correlation of EIF4E3/MEX3A has been defined in breast and lung cancers. Cross-correlation analysis of SSR1/RPL39L was found only in colon and lung cancers, and the cross-correlation of *PPARGC1B/CDKN2A* was found only in colon and breast cancers (Figure 4A–C), R-values of breast, colon and lung data (Supplemental Tables S6–S8).

In conclusion, the plot presents a significant cross-correlation between union RBPs in all cancer types. Other unknown cross-relationships could be accessed through the involvement of other RBPs. However, there was no cross-correlation between *SSR1/RPL39L* in breast cancer, although a cross-correlation between *SSR1/VARS, VARS/PPARGC1B*, and *PPARGC1B/RPL39L* was found.

### 2.4. Survival Analysis

The main aim of this study was to identify survival-associated factors in cancer treatment plans based on gene expression of union RBPs. Therefore, we extracted union RBPs that matched the representative treated patient data, as explained above, from the list of common RBPs to assess the prognostic value of the gene signatures of the 11 RBPs. The gene expression of the union RBPs of each cancer was divided into three subgroups (up/downregulated genes, upregulated genes, and downregulated genes). The RFS or PF of each subgroup for each cancer type was then examined using the Kaplan–Meier estimation method and log-rank test to assess significant differences in the two-group survival curves. The entire breast cancer database includes more than 7830 unique samples out of 55 independent datasets with 5268 RFS patients [12]. The colon cancer database includes 2137 unique samples out of 17 independents cohorts datasets with 1336 RFS patients [13], while the lung cancer database includes 2852 unique samples out of 17 independent cohorts datasets with 1256 PF patients [14]. The results show different contributions between the gene expression and the survival subgroups.

**Figure 5:**
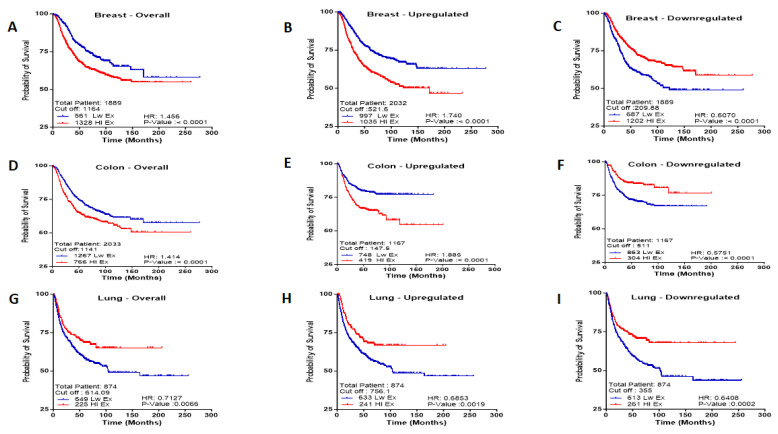
Kaplan–Meier plots representing relapse-free survival (RFS) of breast and colon cancer patients and progression-free survival (PF) of lung cancer patients across the union RBP genes signature subgroups. (**A**,**D**,**G**) All up and downregulated genes, (**B**,**E**,**H**) the upregulated genes and (**C**,**F**,**I**) downregulated genes across breast, colon and lung cancers, respectively.

The list of up/downregulated genes (all genes) including (*CDKN2A, MEX3A, RPL39L, VARS, GSPT1, SNRPE, SSR1, TIA1, PPARGC1B, EIF4E3* and *SMAD9*) were input to breast cancer patients (1889 patients) with 561 low-expression patients and 1328 high-expression patients and a cut-off value for the gene expression of 1164; colon cancer patients (2033 patients) with 1267 low-expression patients, 766 high-expression patients, and a cut-off value for the gene expression of 1141; and lung cancer patients (874 patients) with 649 low-expression patients and 225 high-expression patients and a cut-off value for the gene expression of 614.09. Among all RBP gene signature subgroups, breast, and colon cancer patients with high gene expression had significantly lower survival rates than those with low gene expression. However, lung cancer patients with low gene expression had significantly lower survival rates than those with high gene expression. This indicated 40% more deaths associated with gene high expression in patients who were treated for breast cancer (HR = 1.456, *p*-value < 0.0001), 41% more deaths associated with high gene expression in patients who were treated for colon cancer (HR = 1.41, *p*-value < 0.0001), and 30% more deaths associated with low gene expression for lung cancer (HR = 0.7, *p*-value = 0.007) (Figure 5A,D,G).

The list of upregulated genes (*CDKN2A, MEX3A, RPL39L, VARS, GSPT1, SNRPE, SSR1,* and *TIA1*) were input to breast cancer patients (2032 patients) with 997 low expressed and 1035 high-expression patients with a cut off value between highly and lowly gene expression of 521.6; colon cancer patients (1167 patients) with 748 low-expression patients and 419 high-expression patients and a cut-off value for the gene expression of 147.5; and lung cancer patients (874 patients) with 633 low-expression patients and 241 high-expression patients with a cut-off value for the gene expression of 756.1. Among the upregulated RBP subgroups, breast and colon patients with high gene expression had significantly lower survival rates than those with downregulated gene expression. However, lung cancer patients with low gene expression had significantly lower survival rates than those with downregulated gene expression. This indicated 74% more death associated with high gene expression in patients who were treated for breast cancer (HR = 1.74, *p*-value < 0.0001), 90% more death associated with high gene expression in patients who were treated for colon cancer (HR = 1.89, *p*-value < 0.0001), and 32% more death associated with low gene expression in patients who were treated for lung cancer (HR = 0.685, *p*-value = 0.002) (Figure 5B,E,H).

However, the list of downregulated genes (*PPARGC1B, EIF4E3* and *SMAD9*) was input to breast cancer patients (1889 patients) with 687 low-expression patients and 1202 high-expression patients and a cut-off value for the gene expression of 209.88; colon cancer (1167 patients) with 863 low-expression patients, 304 high-expression patients and a cut-off value for the gene expression of 511; and lung cancer patients (874 patients) with 613 low-expression patients and 261 high-expression patients and a cut-off value for the gene expression of 335. Among the downregulated RBP subgroup, breast, colon, and lung cancer patients with low gene expression had significantly lower survival rates than those with high gene expression. This indicated 40% more deaths associated with low gene expression in patients who were treated for breast cancer (HR = 0.6, *p*-value < 0.0001), 43% more death associated with low gene expression in patients who were treated for colon cancer (HR = 0.6, *p*-value < 0.001), and 36% more death associated with low gene expression in patients who were treated for lung cancer (HR = 0.64, *p*-value = 0.0002) (Figure 5C,F,I).

Hence, upregulation of *CDKN2A, MEX3A, RPL39L, VARS, GSPT1, SNRPE, SSR1,* and *TIA1* is associated with poor survival in breast and colon cancer patients. However, upregulation of *CDKN2A, MEX3A, RPL39L, VARS, GSPT1, SNRPE, SSR1,* and *TIA1 CDKN2A, MEX3A, RPL39L,* and *VARS* and the downregulations of *PPARGC1B, EIF4E3,* and *SMAD9* are associated with poor survival in lung cancer patients. Moreover, the downregulations of *PPARGC1B, EIF4E3,* and *SMAD9* are associated with poor survival in breast, colon, and lung cancer patients.

## 3. Discussion

With the introduction of genomic profiling data and selective molecular-targeted approaches to identify effective therapeutic alternatives, biomarkers have become increasingly important targets for clinical diagnosis and treatment of cancer. Single gene/protein or multigene signature-based assays have been developed to test particular molecular pathway deregulations that direct therapeutic decision making as predictive biomarkers. For example, a clinical trial study showed the influence of a 70-gene signature to improve the prediction of clinical treatment outcomes in women with early-stage breast cancer [15]. Some benefits registered for adjuvant chemotherapy using gene expression test of 21 genes in breast cancer [16]. A six-gene signature for survival prediction in patients with glioblastoma can be used in personalized therapy to promote drug efficiency [17]. Gene expression and computational analysis can be used in adjuvant therapy and gene profiling of non-small cell lung cancer patients at a high risk of relapse [18]. In addition, the effect of CpG-methylation on gene expression is a functional and effective prognostic tool for clear cell renal cell carcinoma (ccRCC) that can add prognostic value to the staging system [19].

Survival and HR analyses are clinical and biostatistical methods used to assess the treatment efficiency in patient groups. Our study focused on identifying survival-associated risk factors and gene signatures of union RBPs across the most common cancers, including breast, colon, and lung cancers. We developed a statistical bioinformatics analysis method based on gene expression, RFS, PF, HR, and *p*-values from the data of treated patients.

Through the process of finding the union RBP gene signature, the master list of RBPs went through data combination, intersection, filtration and classification. During this process, some of the known RBPs were omitted from the list because they did not match our criteria for selection (Figure 1). For instance, *TTP* and *HuR* genes have a high influence on breast cancer [15,16]. The union RBP gene signatures were classified into two subgroups—upregulated and downregulated—that were associated with poor survival conditions. Interestingly, most of the downregulated genes, including *PPARGC1B, EIF4E3,* and *SMAD9*, were classified with poor survival in breast, colon and lung cancer. However, the upregulated genes including *GSPT1, SNRPE, SSR1, CDKN2A, MEX3A, RPL39L, VARS,* and *TIA1* were associated with poor survival in breast and colon cancer but not lung cancer (Table 4). These genes play diverse roles in mRNA metabolism; however, the roles of most of these genes in cancer are not yet identified.

Based on the predicted and experimental STRING data, we identified two signaling pathway-related clusters and non-signaling pathway-related genes in the union RBP gene signatures. The first signaling pathway clusters of the union RBPs were *CDKN2A, GSPT1, SSR1, RPL39L,* and *VARS*. These genes interact and are co-localized and overexpressed in cancer patients. Surprisingly, in this cluster, discrepancies were observed between cellular function, expression correlations, survival-associated RISK factors, and gene HRs. For instance, the overexpression of *CDKN2A, RPL39L*, and *VARS* is associated with poor survival and high risk. However, *GSPT1* and *SSR1* were associated with poor survival and low risk. This may elucidate the competitive relationship in end gene function. Interestingly, crossing the AU-rich database (ARED) with OGS and TS output shows a slightly higher percentage (30%) of OGs than TS genes (28%). This result may highlight that AU-rich motifs have dual functions.

*CDKN2A* is a TS gene that encodes p14 and p16 proteins and is involved in different cellular processes. It showed a high fold induction and was associated with poor survival and a high-risk HR in breast, colon, and lung cancer dataset analyses. According to previous studies, the encoded protein level of *CDKN2A* is almost undetectable. However, it has a dual role in blocking tumor development and cell proliferation, and under oncogenic conditions, its level increases and stimulates p53-dependent and/or -independent pathways [20]. According to STRING analysis, *CDKN2A* binds directly to *GSPT1* (Figure 3D). Based on Curtis analysis, *CDKN2A* had an 11.4% correlation with OGs and only a 4.0% correlation with TS genes (Table 5). *CDKN2A* may have dual functions by controlling the expression or suppression of OGs and TSs.

*RPL39L* is a ribosomal protein paralog that is abundantly expressed in cancer cells and embryonic stem cells. Recently, 2D gel and proteomics analyses suggested that *RPL39L* and other RPs are involved in gene translation [21]. Hypo-methylation of cancer-specific CpG islands (CGIs) and *RPL39L* reactivation are important for the treatment and risk stratification of lung adenocarcinoma [22]. Based on the Curtis analysis, 0.26% correlated with OGs and 4.26% with TS genes, which indicates their role in TS gene expression levels with a strong correlation coefficient, R (0.2–4.26) (Table 5).

*VARS* is one of the 37 aminoacyl-tRNA synthetases (ARSs) [23]. *VARS* and *ARSs* mainly charge tRNA and catalyze the bond between tRNA and designated amino acids. *VARS* mutations are associated with loss of enzymatic activity and the development of a spectrum of global developmental delays, epileptic encephalopathy, and primary or progressive microcephaly [24]. Our results showed that *VARS* binds to the *GST* C-terminal region of the target gene (Table 5). In Curtis, *VARS* showed a 2.4% correlation with OGs and a 0.74% correlation with TS genes (Table 5). The expression level of *VARS* in cancer patients and the high correlation coefficient, R (0.2–0.48), with OGs in contrast to the correlation coefficient, R, of 0.2–0.3 with TS genes may explain the critical role of this gene in catalyzing the bond between the tRNA and amino acid to translate recovery proteins in the treated cancer cells. However, the mechanism requires further investigation.

In eukaryotic cells, the stable G1 to S phase transition protein/eukaryotic release factor (*eRF1*) (*GSPT1*/*eRF3a*) complex is involved in translation termination [25]. *GSPT1* depletion causes cell cycle arrest at the G1 phase via inhibition of the mTOR pathway [26]. There is a statistically significant relationship between the rs4561483 risk genotype and increased *GSPT1* expression in testicular germ cell tumors (TGCTs) [27]. Nicotine and EGF induce genes, including *GSPT1*, to promote the proliferation, invasion, and migration of non-small cell lung cancers, thus enhancing their tumorigenic activity and revealing the central role of the inhibitor of DNA binding/differentiation 1 (ID1) and its downstream targets in facilitating lung cancer progression [28]. In this study, overexpression of *GSPT1* in treated cancer patients was associated with poor survival and low-risk HRs (Table 4). Curtis showed a 2.4% correlation with OGs and a 3.9% correlation with TSs (Table 5).

*SSR1* is part of the *SSR* complex known as *TRAP*. *SSR1*, or the *TRAP*-α subunit, is one of four *TRAP* subunits. The primary function of *TRAP* is protein-specific transport across the endoplasmic reticulum (ER) membrane. Overexpression of *SSR1* in treated cancers may lead to the release of translated genes through the ER rather than to their final destination, where they would usually play specific roles [29]. *TRAP* had a 6.6% correlation with OGs and a 7.0% correlation with TSs, which could be the target genes for *SSR1*. *SSR1* controls TSs by accumulating them in the ER under both pathological and physiological conditions (Table 5). The discrepancy between cellular functions, expression correlations, survival-associated factors, and HRs necessitates further study to understand the cross-functional correlation.

The second signaling pathway cluster of the union RBPs was composed of *TIA1* and *SNRPE*. Both were associated with poor survival and low-risk HRs. The network represents the binding of *TIA1* to the *SNRP* family, showing the critical function of *TIA1* in the complex formation of the *SNRP* family. *TIA1* is an RNA-binding protein that is considered to be a TS and is involved in carcinogenesis. MiR-19a is involved in the destabilization of *TIA1* mRNA by binding directly to its 3′UTR of *TIA1* mRNA [30]. It controls the translation of target genes by binding and co-locating these genes into stress granules (SGs). DNA damage leads to the release of p53 from SGs owing to the dissociation of *TIA1* [31]. *TIA1* mutation is implicated in the delay of SG disassembly and accumulation of non-dynamic SGs, and it is involved in neurodegenerative diseases, such as amyotrophic lateral sclerosis (ALS) [32]. *TIA1* is directly involved in the tau oligomer-mediated pathway. *TIA1* reduces the number and size of SGs, protects against neurodegeneration, and prolongs the survival of transgenic O301S tau mice and tau oligomer aggregation [33]. It is AU-rich in the UTR and has the potential to interact with *Elavl1*/*HuR*. It also recognizes UUUUUGUl RRMX3 binding site motifs. It had a 9.0% correlation with the OGs and a 6.11% correlation with the TGs (Table 5).

*SNRPE* is part of the cellular spliceosome complex that plays a critical role in mRNA maturation [34]. Downregulation of *SNRPE* has been implicated in the dramatic reduction in mTOR mRNA and protein levels and the induction of autophagy [35]. It also plays an oncogenic role in prostate cancer cell proliferation. It directly regulates the androgen receptor (AR), which is involved in cellular proliferation [36]. *SNRPE* overexpression is also associated with lung (adenocarcinoma) prognosis and pathogenesis [37]. Its expression level and role in cell proliferation and invasion in treated cancer patients explain its association with poor survival and low-risk HR. This study found a potential protein–protein interaction between *SNRPE* and *Elavl1*/*HuR*, but this requires further experimental investigation (Table 5).

The non-signaling pathway genes included *MEX3A, PPARGC1B, EIF4E3,* and *SMAD9*. These genes were all associated with poor survival and low-risk HR, except *MEX3A*, which was associated with poor survival and high-risk HR. *MEX3A* is a putative RBP that regulates *CDX2* levels and plays a key role in intestinal differentiation, polarity, and stemness, thereby contributing to cellular homeostasis and carcinogenesis [38]. *MEX3A* reverses the effects of chemotherapy and irradiation by regenerating damaged crypts. This may explain why *MEX3A* levels are higher in treated patients than in untreated patients and why treated patients have a low-risk HR when undergoing this type of treatment [39]. *MEX3A* contains an AU-rich motif at the UTR site that binds to the KHx2, znf, and CCCHx1 binding sites of target genes.

The *PGC1B* is encoded by the *PPARGC1B* gene. AMP-activated kinase promotes aberrant *PGC1B* expression in human colon cancer cells [40]. *PGC1A* and *PGC1B* methylation are early cancer risk biomarkers that recognize the *RRMK1* binding site motif [41]. Here, we showed a correlation between *PGC1B* downregulation and poor survival, which was associated with a low risk of death and cancer relapse (Table 5). Therefore, further investigations are required.

*EIF4E3* belongs to the *EIF4E* protein family that includes *EIF4E* proteins 1, 2, and 3. They play essential roles in the initiation of protein translation, which occurs during mRNA metabolism, proliferation, survival, invasion and metastasis [42]. In particular, *EIF4E3* binds to the positively charged m^7^G cap to compete with other factors and functions as TS. The reduction in *EIF4E3* in cancers with high *EIF4E* levels suggests that *EIF4E3* is a clinically relevant inhibitory mechanism in some malignancies [43]. In parallel survival analysis of patients with breast cancer, overexpression of certain genes, including *EIF4E3*, improved survival rates [44]. The phosphorylation of *EIF4E1* has been implicated in the initiation of oncogenic mRNA translation. Enhanced *EIF4E3* expression competes with *EIF4E1* and suppresses *EIF4E1*-driven translation, revealing a novel role for *EIF4E3* in translation imitation biology [45]. This study showed a 7.0% correlation with OGs and an 8.0% correlation with TSs. This competition may explain the dual role of *EIF4E3* in controlling gene induction under certain physiological and pathological conditions.

*SMAD9* belongs to a family of nine proteins (*SMAD* 1-9), which are divided into three subgroups: receptor *SMAD* proteins (R-*SMADs*), which comprise *SMADs* 1, 2, 3, 5, and 9 (8). Cofactor *SMAD* proteins (Co-*SMADs*), comprising only *SMAD4*, and inhibitor *SMAD* proteins (I-*SMADs*), comprising *SMADs* 6 and 7. *SMADs* are transcriptional regulators of intracellular TGF-β signaling. The major role of *SMAD9* is to suppress target gene transcription by competing with *SMAD1* to form a DNA-binding complex that binds DNA [46]. We found that downregulation of SMAD9 in treated cancer patients was associated with poor survival and a low risk of death.

*MEX3A, GSPT1, SSR1, TIA1, PGC1B, EIF4E3,* and *SMAD9* contained AU-rich elements (AREs) at the 3′UTR mRNA site (Table 5). AREs are cis-acting instability and translation inhibition elements present in the 3′-UTR and introns of most inducible genes [47,48]. *HuR/Elavl1* is associated with intronic AREs that are involved in the posttranscriptional regulation of more than half of the human genes [47]. Abnormal ARE-mediated post-transcriptional control is associated with several abnormal cellular processes underlying carcinogenesis. RBPs, *TTP, and HuR* have antagonistic roles in mRNA regulation in metastatic breast cancer. *TTP* destabilizes mRNA and suppresses protein translation, whereas *HuR* is an mRNA-binding and translation-promoting component. Low *TTP/HuR* mRNA ratios are associated with poor survival in breast cancer patients and high levels of mitotic ARE-mRNA signatures, highlighting the role of AREs and their binding proteins in cancer [49]. A review article illustrates the association of dysfunctional AU-rich RBPs in human cancers [50]. Another review article proposes the influence of competition between stabilizing and destabilizing AU-rich RBPs in mRNA regulation [51]. Further investigation is required to better understand the relationship between the *TTP/HuR* ratio and stability of *MEX3A, GSPT1, SSR1, TIA1, PGC1B, EIF4E3,* and *SMAD9* expression.

In summary, the computational bioinformatics analysis tool on patient data identified the union RBP gene signatures and their association with survival data in breast, colon, and lung cancers. These RBPs might be involved in the occurrence, development, invasion, metastasis, and drug resistance of cancer cells.

In conclusion, the roles of union RBP genes in cancer development and drug resistance remain unclear. Further in vitro and in vivo studies are required to verify their functions and contributions to cancer development, drug resistance, and the influence of ethnicity, sex, and epigenetic diversity on drug efficiency. In particular, downregulated union RBP gene selection results illustrate that our extended subtyping framework, by combining subtyping and subtype-specific biomarkers, may lead to improved patient prognostication, form a strong basis for future studies, and potentially be applied as a personalized diagnostic test panel for routine laboratory tests.

## 4. Materials and Methods

### 4.1. Availability of Data and Union RBP Intersection Master List and Gene Clustering

A total of 3302 genes generated and/or analyzed in this study were obtained and compiled from three different RNA databases: RBPome, Census, and RBPDB. RBPome (a catalogue of 1344 experimentally confirmed RBP genes) was downloaded from a Supplemental Table published in https://www.nature.com/articles/nrg3813 (accessed on 22 January 2025) (Supplemental Table S1), Census (a manually curated RBPs of 1542 genes) was downloaded from a Supplemental Table published in https://pubmed.ncbi.nlm.nih.gov/25365966/ (accessed on 22 January 2025) (Supplemental Table S2), and the RBPDB database (experimental RBPs with known RNA-binding domains of 416 genes that were manually curated from the literature) was downloaded from http://rbpdb.ccbr.utoronto.ca (accessed on 22 January 2025) (Supplemental Table S3) [8,9].

### 4.2. Functional and Pathway Enrichment Analysis of Union RBPs

To comprehensively analyze the biological functions of the union RBPs, we used the Protein Analysis Through Evolutionary Relationships (PANTHER-19.0) gene ontology (GO) software website to visualize the biological and molecular processes and integration of the genes. Pie graphs were constructed using GraphPad Prism, version 6.

### 4.3. Protein–Protein Interaction Network Construction and Interrelation Analysis Between Pathways

STRING version 12 (https://string-db.org) (accessed on 22 January 2025) was used to evaluate the current interaction networks and experiments on protein–protein interactions. The interaction networks of these proteins were visualized by executing a list of 11 protein identifiers in multiple searches and selecting Homo sapiens as the organism. The protein network was analyzed to determine the interactions at the protein level.

### 4.4. RBP Cross-Correlation

The expression of 11 genes was downloaded from Oncomine, TCGA Breast, TCGA Colon, and Yokohama Lung. The expression of 389, 102, and 226 diseased genes for breast, colon, and lung cancer, respectively, was collected, and multivariate correlations across multiple genes were calculated using SAS Institute Inc. 2013, Cary, NC, USA (SAS^®^ Enterprise Guide™ 6.1). The correlation values and *p*-values were entered into GraphPad Prism version 6.05 to construct a volcano plot.

### 4.5. RBP Co-Expression with Oncogene/Tumor Suppressor Genes

A co-expression analysis was performed using Oncomine on the genes in the Curtis breast database, a comprehensive database that includes information on many patients. A list of correlated genes was downloaded for each gene of interest in each cancer type. This list was searched for matching oncogene (OG) and tumor suppressor (TS) genes. The OG database (378 genes) was downloaded from http://ongene.bioinfo-minzhao.org/ (accessed on 22 January 2025) and the TS database (540 genes) from https://bioinfo.uth.edu/TSGene/ (accessed on 22 January 2025). To obtain statistically significant correlations, the list of matched OG and TS genes was filtered out to extract only genes with correlation coefficients R ≥ 0.20.

### 4.6. Kaplan–Meier Plot

Kaplan–Meier survival analyses were performed using the Kaplan–Meier Plotter (http://kmplot.com/analysis/) (accessed on 22 January 2025), a comprehensive dataset for survival analysis that includes the cross-normalized expression data of 54,675 genes in 4142 breast cancer patients. The database was built using gene expression and survival data from the European Genome-Phenome Archive (EGA) and Gene Expression Omnibus (GEO) repository. Recurrence-free survival (RFS) was determined using gene cluster analysis. Associations between gene expression and patient survival were assessed using the Kaplan–Meier method (log-rank test, GraphPad 6.0) assessed associations between gene expression and patient survival. The percentile threshold algorithm (25) was used to determine the optimal cut-off of the members of the RBP cluster. The best probe set was selected if multiple probe sets measured the same gene, to ensure the optimal probe set for each gene. Hazard ratios (HRs) and *p*-values were determined using the Cox proportional hazards analysis. KMP calculates cutoff values between the upper and lower quartiles for selected genes (Union RBPs) across patient data and employs the Benjamini–Hochberg method to control the false discovery rate (FDR) during multiple hypothesis testing. The cutoff values were selected to achieve the highest significance while maintaining a low FDR. If multiple cutoff values had equal significance, the one with the highest hazard ratio (HR) was chosen for the final analysis [12,13,14].

## Figures and Tables

**Table 1:** Details of the gene expression microarray datasets downloaded from of Oncomine.

Dataset	No Samples	No Array Genes	Portal	Platform	Reference
TCGA, Breast	593	20,423	Oncomine	Not defined	No associated paper 3 June 2013
TCGA, Colorectal	237	20,423	Oncomine	Not Defined	[10]
Okayama, Lung	246	19,574	Oncomine	Human Genome U133 Plus 2.0 Array	[11]

**Table 2:** Union RBP genes clustering: Tables (A–**C**) represent the six clusters including the number of genes, gene expression fold change, HR and *p*-values of each cluster across (**A**) breast, (**B**) colon and (**C**) lung cancer.

A: Breast				
**Cluster**	**Count**	**Fold**	**HR**	***p*-Value**
**1**	13	−3.0899	0.7487	0.0053
**2**	4	−3.0786	1.1749	0.0531
**3**	15	1.8787	0.7468	0.0018
**4**	7	2.4200	1.0572	0.0715
**5**	18	2.2835	1.4526	0.0001
**6**	1	1.8811	1.0600	0.4300
B: Colon				
**Cluster**	**Count**	**Fold**	**HR**	***p*-Value**
**1**	8	−3.4020	0.6750	0.0347
**2**	12	−3.0220	1.6226	0.0090
**3**	5	−2.8213	0.9812	0.3408
**4**	21	1.9641	0.6320	0.0042
**5**	5	1.7852	0.8115	0.1130
**6**	7	2.4175	1.3280	0.0573
C: Lung				
**Cluster**	**Count**	**Fold**	**HR**	***p*-Value**
**1**	11	−2.2714	0.5686	0.0008
**2**	17	1.7615	0.6425	0.0046
**3**	3	−2.0165	0.8606	0.2137
**4**	1	1.5968	1.1200	0.2700
**5**	2	−2.0638	1.7699	0.0002
**6**	24	1.9161	1.5380	0.0086

**Table 3:** The union RBP genes class contributions: Class 1 shows genes with criteria of HR value > 1 and FI > 1.5. Class 2 shows genes with criteria of HR value < 1 and FI > 1.5. Class 3 shows genes with criteria of HR value > 1 and FI < 1.5. Class 4 shows genes with criteria of HR value < 1 and FI < −1.5.

	Up-Regulated	Down-Regulated
	Breast/Colon/Lung	Breast/Colon/Lung
	(FI > 1.5)	(FI < 1.5)
**High-risk (HR > 1)**	**(Class 1)**	**(Class 3)**
	MEX3A	NA
	CDKN2A	
	RPL39L	
	VARS	
**Low-risk (HR < 1)**	**(Class 2)**	**(Class 4)**
	GSPT1	PPARGC1B
	SNRPE	EIF4E3
	SSR1	SMAD9
	TIA1	

**Table 4:** The mean expression levels of union RBP genes across the three types of cancers along with their HR values and their consensus targets.

	Breast	Colon	Lung	Average Fold Induction	Average HR-Value	Cosensus	Faunction
	FI	FI	FI	Breast, Colon & Lung	Breast, Colon & Lung	Target	
***CDKN2A***	2.317773	3.7912054	2.7925382	2.967172	1.36098	unknown	TS that encoding p14 and p16 which are involved in different cellular processes.
***MEX3A***	2.8456798	4.859792	1.980403	3.228625	1.500737	mRNA	Putative RBP involve in polarity and stremness that contributes with cellular homeostasis and carcinogenesis.
***RPL39L***	1.6922513	1.72875	2.2474358	1.889479	1.49	ribosome	Ribosomal Protein Paralogs that are involved in gene transiations.
***VARS***	1.6565965	1.5851151	1.5416017	1.594438	1.31837	tRNA	Changing and catalysing the bond between tRNA and designated amino acid.
***GSPT1***	1.7117828	1.804728	1.636707	1.717739	0.613333	mRNA	Termination of protein translation.
***SNRPE***	1.8730097	1.96655	1.5880028	1.809188	0.686667	snRNA	Cellulare splicesome complex that involve in mRNA maturation process.
***SSR1***	1.759067	1.5236051	1.7405431	1.674405	0.683333	unknown	Proteins-specific transportation across ER membrane.
***TIA1***	1.5232474	1.7893108	1.5973161	1.636625	0.66	mRNA	Cosider as a TS that involved in controlling the translation and co-localization of target genes in SGs.
***PPARGC1B***	−1.9268708	−3.055817	−2.537169	−2.50662	0.6805	unknown	unknown.
***EIF4E3***	−2.5564525	−4.619756	−1.8139602	−2.99672	0.605903	mRNA	Play essentials roles in initiation the protein translation that are involved in mRNA metabolism.
***SMAD9***	−2.2608936	−2.207682	−2.8138292	−2.42747	0.663987	putative miRNA	Belong to receptor SMAD protein complex, binds to DNA in process of suppressing of target gene transcription.

**Table 5:** Union RBP genes summary; binding site, AU-rich and correlation to oncogenes and tumor suppression genes. ** There is no available correlation data in the Curtis database.

Symbol	Desccriptions	Motif	Binding Site	STRING:PPI with elavl1/TTP	% ONCO Correlation	% TS Correlation
**CDKN2A**	Cyclin-dependent kinase inhibitor 2A	unknown	N/A	none	11.37%	4.07%
**MEX3A**	RNA-binding protein MEX3A	AURICH motif	KHx2; Znf_CCCHx1	none	**	**
**RPL39L**	Ribosomal protein L39 like	unknown	N/A	none	0.26%	4.26%
**VARS**	Valine--tRNA ligase; Aminoacyl tRNA synthetases, Class I	unknown	GST c-terminal	none	2.38%	0.74%
**GSPT1**	Eukaryotic peptide chain release factor GTP-binding subunit ERF3A	AURICH motif	N/A	none	2.38%	3.89%
**SNRPE**	Small nuclear ribonucleoprotein E	unknown	LSmx1	elavl1	**	**
**SSR1**	Translocon-associated protein subunit alpha	AURICH motif	N/A	none	6.61%	7.04%
**TIA1**	Nucleolysin TIA-1 isoform p40	AURICH motif	UUUUUGU / RRMX3	elavl1	8.99%	6.11%
**PPARGC1B**	Peroxisome proliferator-activated receptor gamma coactivator 1-beta	AURICH motif	RRMx1	none	**	**
**EIF4E3**	karyotic translation initiation factor 4E type 3	AURICH motif	N/A	none	7.14%	8.15%
**SMAD9**	Mothers against decapentaplegic homolog 9	AURICH motif	N/A	none	**	**

## Data Availability

The authors confirm that the data used in this study are either cited or acknowledged within the article and/or its Supplementary Material.

## Supplementary Materials

The supporting information can be downloaded at: https://www.mdpi.com/article/10.3390/ijms26031017/s1.

## Abbreviations

AREs: AU-rich elements; RBPs: RNA-binding proteins; HR: hazard ratios; PF: Progression-free survival; RFS: Relapse-free survival; TRAP: Translocon-associated proteincomplex; OG: Oncogenes; TS: Tumor suppressor genes; CDKN2A: cyclin-dependent kinase inhibitor 2A; MEX3A mex-3 RNA binding family member A; RPL39L ribosomal protein L39 like; GSPT1: G1 to S phase transition 1; SNRPE small nuclear ribonucleoprotein polypeptide E; SSR1 signal sequence receptor subunit 1; TIA1: T cell intracellular antigen 1; PPARGC1B: peroxisome proliferator-activated receptor gamma co-activator 1 beta; EIF4E3: eukaryotic translation initiation factor 4E family member 3; SMAD9: SMAD family member 9; and VARS: Valine cytoplasmic-localized aminoacyl-tRNA synthetase. TTP (ZFP36 family of RBPs) or Tristetraprolin. HUR is known Hu Antigen R or ELAVL1 Embryonic Lethal, Abnormal Vision, Drosophila Like 1.
