# Supplementary material for: A Computational Recognition Analysis of Promising Prognostic Biomarkers in Breast, Colon and Lung Cancer Patients
Source: Int J Mol Sci. 2025 Jan 25;26(3):1017. doi: 10.3390/ijms26031017 (PMC11817791; doi:10.3390/ijms26031017)
Supplement: Supplementary file 1 [file ijms-26-01017-s001.zip › ijms-3375022-supplementary.pdf]

Supplemental Table S1	
RBPOME database	
**Gene Name**	**Gene ID**
A1CF	ENSG00000148584
AATF	ENSG00000108270
ABCF1	ENSG00000204574
ABT1	ENSG00000146109
AC004381.6	ENSG00000005189
AC104534.3	ENSG00000268083
ACAA2	ENSG00000167315
ACIN1	ENSG00000100813
ACO1	ENSG00000122729
ACTN1	ENSG00000072110
ACTN4	ENSG00000130402
ADAR	ENSG00000160710
ADARB1	ENSG00000197381
ADARB2	ENSG00000185736
ADD1	ENSG00000087274
ADK	ENSG00000156110
AEN	ENSG00000181026
AGO1	ENSG00000092847
AGO2	ENSG00000123908
AGO3	ENSG00000126070
AHNAK	ENSG00000124942
AKAP1	ENSG00000121057
AKAP17A	ENSG00000197976
AKAP8	ENSG00000105127
AKAP8L	ENSG00000011243
ALDH18A1	ENSG00000059573
ALDH6A1	ENSG00000119711
ALDOA	ENSG00000149925
ALG13	ENSG00000101901
ALKBH5	ENSG00000091542
ALKBH8	ENSG00000137760
ALYREF	ENSG00000183684
ANKHD1	ENSG00000131503
ANKRD17	ENSG00000132466
ANP32A	ENSG00000140350
ANP32B	ENSG00000136938
ANXA11	ENSG00000122359
ANXA2	ENSG00000182718
ANXA7	ENSG00000138279
APEH	ENSG00000164062
APEX1	ENSG00000100823
API5	ENSG00000166181
APOBEC3B	ENSG00000179750

APOBEC3C	ENSG00000244509
APOBEC3F	ENSG00000128394
APOBEC3H	ENSG00000100298
APTX	ENSG00000137074
AQR	ENSG00000021776
ARCN1	ENSG00000095139
ARF1	ENSG00000143761
ARHGEF1	ENSG00000076928
ARL6IP4	ENSG00000182196
ASCC1	ENSG00000138303
ASCC3	ENSG00000112249
ASS1	ENSG00000130707
ATP5A1	ENSG00000152234
ATP5C1	ENSG00000165629
ATP5J2-PTCD1	ENSG00000248919
ATXN2	ENSG00000204842
ATXN2L	ENSG00000168488
BAG4	ENSG00000156735
BCCIP	ENSG00000107949
BCLAF1	ENSG00000029363
BICC1	ENSG00000122870
BMS1	ENSG00000165733
BOLL	ENSG00000152430
BOP1	ENSG00000170727
BRIX1	ENSG00000113460
BST2	ENSG00000130303
BTF3	ENSG00000145741
BUD13	ENSG00000137656
BYSL	ENSG00000112578
BZW1	ENSG00000082153
BZW2	ENSG00000136261
C11orf31	ENSG00000211450
C11orf68	ENSG00000175573
C12orf43	ENSG00000157895
C14orf166	ENSG00000087302
C14orf93	ENSG00000100802
C15orf52	ENSG00000188549
C16orf80	ENSG00000070761
C17orf85	ENSG00000074356
C19orf47	ENSG00000160392
C1orf131	ENSG00000143633
C1orf204	ENSG00000188004
C1orf35	ENSG00000143793
C1orf52	ENSG00000162642
C2orf15	ENSG00000241962
C4BPA	ENSG00000123838
C7orf50	ENSG00000146540

C7orf55-LUC7L2	ENSG00000146963
C9orf114	ENSG00000198917
CACTIN	ENSG00000105298
CALR	ENSG00000179218
CANX	ENSG00000127022
CAPRIN1	ENSG00000135387
CARHSP1	ENSG00000153048
CASC3	ENSG00000108349
CAST	ENSG00000153113
CCAR1	ENSG00000060339
CCAR2	ENSG00000158941
CCBL2	ENSG00000137944
CCDC108	ENSG00000181378
CCDC124	ENSG00000007080
CCDC137	ENSG00000185298
CCDC47	ENSG00000108588
CCDC59	ENSG00000133773
CCDC86	ENSG00000110104
CCDC9	ENSG00000105321
CCT3	ENSG00000163468
CCT4	ENSG00000115484
CCT6A	ENSG00000146731
CD3EAP	ENSG00000117877
CDC40	ENSG00000168438
CDC42EP4	ENSG00000179604
CDC5L	ENSG00000096401
CDK11B	ENSG00000248333
CDK13	ENSG00000065883
CDKN2A	ENSG00000147889
CEBPZ	ENSG00000115816
CELF1	ENSG00000149187
CELF2	ENSG00000048740
CELF3	ENSG00000159409
CELF4	ENSG00000101489
CELF5	ENSG00000161082
CELF6	ENSG00000140488
CGGBP1	ENSG00000163320
CHCHD1	ENSG00000172586
CHD2	ENSG00000173575
CHD3	ENSG00000170004
CHERP	ENSG00000085872
CHTOP	ENSG00000160679
CIRBP	ENSG00000099622
CIRH1A	ENSG00000141076
CISD2	ENSG00000145354
CKAP4	ENSG00000136026
CLASRP	ENSG00000104859

CLK3	ENSG00000179335
CLNS1A	ENSG00000074201
CLTC	ENSG00000141367
CMSS1	ENSG00000184220
CNBP	ENSG00000169714
CNN3	ENSG00000117519
CNOT1	ENSG00000125107
CNOT4	ENSG00000080802
COA6	ENSG00000168275
COL14A1	ENSG00000187955
CORO1A	ENSG00000102879
CPEB1	ENSG00000214575
CPEB2	ENSG00000137449
CPEB3	ENSG00000107864
CPEB4	ENSG00000113742
CPNE3	ENSG00000085719
CPSF4	ENSG00000160917
CPSF4L	ENSG00000187959
CPSF6	ENSG00000111605
CPSF7	ENSG00000149532
CRKL	ENSG00000099942
CRNKL1	ENSG00000101343
CS	ENSG00000062485
CSDC2	ENSG00000172346
CSDE1	ENSG00000009307
CSNK1E	ENSG00000213923
CSRP1	ENSG00000159176
CSTB	ENSG00000160213
CSTF1	ENSG00000101138
CSTF2	ENSG00000101811
CSTF2T	ENSG00000177613
CSTF3	ENSG00000176102
CTD-2369P2.12	ENSG00000267303
CTNNA1	ENSG00000044115
CWC15	ENSG00000150316
CXorf57	ENSG00000147231
DAP3	ENSG00000132676
DARS	ENSG00000115866
DAZ1	ENSG00000188120
DAZ2	ENSG00000205944
DAZ3	ENSG00000187191
DAZ4	ENSG00000205916
DAZAP1	ENSG00000071626
DAZL	ENSG00000092345
DBR1	ENSG00000138231
DCAF13	ENSG00000164934
DCD	ENSG00000161634

DCN	ENSG00000011465
DDX1	ENSG00000079785
DDX10	ENSG00000178105
DDX17	ENSG00000100201
DDX18	ENSG00000088205
DDX21	ENSG00000165732
DDX23	ENSG00000174243
DDX24	ENSG00000089737
DDX27	ENSG00000124228
DDX28	ENSG00000182810
DDX31	ENSG00000125485
DDX39A	ENSG00000123136
DDX39B	ENSG00000198563
DDX39B(SUPERCONTIG)	ENSG00000215425
DDX3X	ENSG00000215301
DDX41	ENSG00000183258
DDX42	ENSG00000198231
DDX43	ENSG00000080007
DDX46	ENSG00000145833
DDX47	ENSG00000213782
DDX49	ENSG00000105671
DDX5	ENSG00000108654
DDX50	ENSG00000107625
DDX51	ENSG00000185163
DDX52	ENSG00000141141
DDX53	ENSG00000184735
DDX54	ENSG00000123064
DDX55	ENSG00000111364
DDX56	ENSG00000136271
DDX6	ENSG00000110367
DEK	ENSG00000124795
DGKQ	ENSG00000145214
DHX15	ENSG00000109606
DHX16	ENSG00000204560
DHX29	ENSG00000067248
DHX30	ENSG00000132153
DHX33	ENSG00000005100
DHX34	ENSG00000134815
DHX36	ENSG00000174953
DHX38	ENSG00000140829
DHX57	ENSG00000163214
DHX8	ENSG00000067596
DHX9	ENSG00000135829
DIAPH1	ENSG00000131504
DIDO1	ENSG00000101191
DIEXF	ENSG00000117597
DIMT1	ENSG00000086189

DIS3	ENSG00000083520
DKC1	ENSG00000130826
DMGDH	ENSG00000132837
DNAJC17	ENSG00000104129
DNAJC2	ENSG00000105821
DNAJC21	ENSG00000168724
DNAJC8	ENSG00000126698
DNM1	ENSG00000106976
DNTTIP2	ENSG00000067334
DPPA5	ENSG00000203909
DROSHA	ENSG00000113360
DSP	ENSG00000096696
DUS3L	ENSG00000141994
DUSP11	ENSG00000144048
DUSP14	ENSG00000161326
DUT	ENSG00000128951
DYNC1H1	ENSG00000197102
DYNC1LI1	ENSG00000144635
DZIP3	ENSG00000198919
EBNA1BP2	ENSG00000117395
EDF1	ENSG00000107223
EED	ENSG00000074266
EEF1A1	ENSG00000156508
EEF1G	ENSG00000254772
EEF2	ENSG00000167658
EFTUD2	ENSG00000108883
EIF1	ENSG00000173812
EIF1AX	ENSG00000173674
EIF1B	ENSG00000114784
EIF2AK2	ENSG00000055332
EIF2D	ENSG00000143486
EIF2S1	ENSG00000134001
EIF2S2	ENSG00000125977
EIF3A	ENSG00000107581
EIF3B	ENSG00000106263
EIF3C	ENSG00000184110
EIF3CL	ENSG00000205609
EIF3D	ENSG00000100353
EIF3E	ENSG00000104408
EIF3G	ENSG00000130811
EIF3H	ENSG00000147677
EIF3L	ENSG00000100129
EIF4A1	ENSG00000161960
EIF4A2	ENSG00000156976
EIF4A3	ENSG00000141543
EIF4B	ENSG00000063046
EIF4E	ENSG00000151247

EIF4E2	ENSG00000135930
EIF4ENIF1	ENSG00000184708
EIF4G1	ENSG00000114867
EIF4G2	ENSG00000110321
EIF4G3	ENSG00000075151
EIF4H	ENSG00000106682
EIF5	ENSG00000100664
EIF5A	ENSG00000132507
EIF5A2	ENSG00000163577
EIF5B	ENSG00000158417
ELAC2	ENSG00000006744
ELAVL1	ENSG00000066044
ELAVL2	ENSG00000107105
ELAVL3	ENSG00000196361
ELAVL4	ENSG00000162374
EMG1	ENSG00000126749
ENO1	ENSG00000074800
ENOX1	ENSG00000120658
ENOX2	ENSG00000165675
EPPK1	ENSG00000227184
ERAL1	ENSG00000132591
ERH	ENSG00000100632
ERI3	ENSG00000117419
ESF1	ENSG00000089048
ESRP1	ENSG00000104413
ESRP2	ENSG00000103067
ETF1	ENSG00000120705
EWSR1	ENSG00000182944
EXOSC1	ENSG00000171311
EXOSC10	ENSG00000171824
EXOSC2	ENSG00000130713
EXOSC4	ENSG00000178896
EXOSC6	ENSG00000223496
EXOSC9	ENSG00000123737
EZR	ENSG00000092820
FAM103A1	ENSG00000169612
FAM120A	ENSG00000048828
FAM120C	ENSG00000184083
FAM133B	ENSG00000234545
FAM207A	ENSG00000160256
FAM208A	ENSG00000163946
FAM32A	ENSG00000105058
FAM46A	ENSG00000112773
FAM50A	ENSG00000071859
FAM98A	ENSG00000119812
FAM98B	ENSG00000171262
FARSA	ENSG00000179115

FARSB	ENSG00000116120
FASN	ENSG00000169710
FASTKD1	ENSG00000138399
FASTKD2	ENSG00000118246
FASTKD3	ENSG00000124279
FASTKD5	ENSG00000215251
FAU	ENSG00000149806
FBL	ENSG00000105202
FBLL1	ENSG00000188573
FBRSL1	ENSG00000112787
FCF1	ENSG00000119616
FDPS	ENSG00000160752
FIP1L1	ENSG00000145216
FKBP3	ENSG00000100442
FKBP4	ENSG00000004478
FLNA	ENSG00000196924
FLNB	ENSG00000136068
FLYWCH2	ENSG00000162076
FMR1	ENSG00000102081
FNDC3A	ENSG00000102531
FNDC3B	ENSG00000075420
FRG1	ENSG00000109536
FSCN1	ENSG00000075618
FTSJ3	ENSG00000108592
FUBP1	ENSG00000162613
FUBP3	ENSG00000107164
FUS	ENSG00000089280
FXR1	ENSG00000114416
FXR2	ENSG00000129245
FYTTD1	ENSG00000122068
G3BP1	ENSG00000145907
G3BP2	ENSG00000138757
GANAB	ENSG00000089597
GAPDH	ENSG00000111640
GAR1	ENSG00000109534
GCN1L1	ENSG00000089154
GDI2	ENSG00000057608
GEMIN5	ENSG00000082516
GFM1	ENSG00000168827
GIGYF2	ENSG00000204120
GLRX3	ENSG00000108010
GLTSCR2	ENSG00000105373
GNB2L1	ENSG00000204628
GNL2	ENSG00000134697
GNL3	ENSG00000163938
GNL3L	ENSG00000130119
GOLGB1	ENSG00000173230

GOT2	ENSG00000125166
GPATCH4	ENSG00000160818
GPATCH8	ENSG00000186566
GPX4	ENSG00000167468
GRB2	ENSG00000177885
GRN	ENSG00000030582
GRSF1	ENSG00000132463
GRWD1	ENSG00000105447
GSPT1	ENSG00000103342
GSPT2	ENSG00000189369
GTF2E2	ENSG00000197265
GTF2F1	ENSG00000125651
GTF3A	ENSG00000122034
GTPBP1	ENSG00000100226
GTPBP10	ENSG00000105793
GTPBP4	ENSG00000107937
H1F0	ENSG00000189060
H1FX	ENSG00000184897
HADHB	ENSG00000138029
HARS2	ENSG00000112855
HDAC2	ENSG00000196591
HDGF	ENSG00000143321
HDLBP	ENSG00000115677
HEATR1	ENSG00000119285
HEATR6	ENSG00000068097
HELZ	ENSG00000198265
HELZ2	ENSG00000130589
HERC5	ENSG00000138646
HEXIM1	ENSG00000186834
HIST1H1B	ENSG00000184357
HIST1H1C	ENSG00000187837
HIST1H1D	ENSG00000124575
HIST1H1E	ENSG00000168298
HIST1H4H	ENSG00000158406
HIST2H4B	ENSG00000182217
HK2	ENSG00000159399
HLA-A	ENSG00000235657
HLTF	ENSG00000071794
HMCES	ENSG00000183624
HMGB1	ENSG00000189403
HMGB2	ENSG00000164104
HMGB3	ENSG00000029993
HMGN2	ENSG00000198830
HMGN5	ENSG00000198157
HNRNPA0	ENSG00000177733
HNRNPA1	ENSG00000135486
HNRNPA1L2	ENSG00000139675

HNRNPA1P36	ENSG00000231942
HNRNPA1P7	ENSG00000215492
HNRNPA2B1	ENSG00000122566
HNRNPA3	ENSG00000170144
HNRNPAB	ENSG00000197451
HNRNPC	ENSG00000092199
HNRNPCL1	ENSG00000179172
HNRNPD	ENSG00000138668
HNRNPDL	ENSG00000152795
HNRNPF	ENSG00000169813
HNRNPH1	ENSG00000169045
HNRNPH2	ENSG00000126945
HNRNPH3	ENSG00000096746
HNRNPK	ENSG00000165119
HNRNPL	ENSG00000104824
HNRNPLL	ENSG00000143889
HNRNPM	ENSG00000099783
HNRNPR	ENSG00000125944
HNRNPU	ENSG00000153187
HNRNPUL1	ENSG00000105323
HNRNPUL2	ENSG00000214753
HOXB6	ENSG00000108511
HRSP12	ENSG00000132541
HSD17B10	ENSG00000072506
HSP90AA1	ENSG00000080824
HSP90AB1	ENSG00000096384
HSP90B1	ENSG00000166598
HSPA1A	ENSG00000204389
HSPA1B	ENSG00000204388
HSPA5	ENSG00000044574
HSPA8	ENSG00000109971
HSPA9	ENSG00000113013
HSPB1	ENSG00000106211
HSPD1	ENSG00000144381
HSPE1	ENSG00000115541
HSPG2	ENSG00000142798
HTATSF1	ENSG00000102241
HUWE1	ENSG00000086758
IBA57	ENSG00000181873
IFI16	ENSG00000163565
IFIT2	ENSG00000119922
IFIT5	ENSG00000152778
IGF2BP1	ENSG00000159217
IGF2BP2	ENSG00000073792
IGF2BP3	ENSG00000136231
ILF2	ENSG00000143621
ILF3	ENSG00000129351

IMMT	ENSG00000132305
IMP3	ENSG00000177971
IMP4	ENSG00000136718
IPO5	ENSG00000065150
IREB2	ENSG00000136381
ISG20L2	ENSG00000143319
ISY1	ENSG00000240682
ISY1-RAB43	ENSG00000261796
JUN	ENSG00000177606
KCTD12	ENSG00000178695
KHDRBS1	ENSG00000121774
KHDRBS2	ENSG00000112232
KHDRBS3	ENSG00000131773
KHSRP	ENSG00000088247
KIAA0020	ENSG00000080608
KIAA0430	ENSG00000166783
KIAA1324	ENSG00000116299
KIAA1429	ENSG00000164944
KIF1C	ENSG00000129250
KIN	ENSG00000151657
KM-PA-2	ENSG00000204775
KMT2C	ENSG00000055609
KNOP1	ENSG00000103550
KPNA2	ENSG00000182481
KPNB1	ENSG00000108424
KRI1	ENSG00000129347
KRR1	ENSG00000111615
KRT18	ENSG00000111057
KTN1	ENSG00000126777
KXD1	ENSG00000105700
L1TD1	ENSG00000240563
L3MBTL3	ENSG00000198945
LARP1	ENSG00000155506
LARP1B	ENSG00000138709
LARP4	ENSG00000161813
LARP4B	ENSG00000107929
LARP6	ENSG00000166173
LARP7	ENSG00000174720
LAS1L	ENSG00000001497
LBR	ENSG00000143815
LEMD3	ENSG00000174106
LENG9	ENSG00000182909
LGALS1	ENSG00000100097
LGALS3	ENSG00000131981
LIN28A	ENSG00000131914
LIN28B	ENSG00000187772
LLPH	ENSG00000139233

LRP1	ENSG00000123384
LRPPRC	ENSG00000138095
LRRC47	ENSG00000130764
LRRC59	ENSG00000108829
LSG1	ENSG00000041802
LSM1	ENSG00000175324
LSM10	ENSG00000181817
LSM11	ENSG00000155858
LSM14A	ENSG00000257103
LSM14B	ENSG00000149657
LSM2	ENSG00000204392
LSM3	ENSG00000170860
LSM4	ENSG00000130520
LSM5	ENSG00000106355
LSM6	ENSG00000164167
LSM7	ENSG00000130332
LSMD1	ENSG00000183011
LTA4H	ENSG00000111144
LUC7L	ENSG00000007392
LUC7L2	ENSG00000269955
LUC7L3	ENSG00000108848
LYAR	ENSG00000145220
MACF1	ENSG00000127603
MAGOH	ENSG00000162385
MAGOH2	ENSG00000264176
MAK16	ENSG00000198042
MAP4	ENSG00000047849
MAPRE1	ENSG00000101367
MARK2	ENSG00000072518
MATR3	ENSG00000015479
MAZ	ENSG00000103495
MBNL1	ENSG00000152601
MBNL2	ENSG00000139793
MBNL3	ENSG00000076770
MCAT	ENSG00000100294
MCM3AP	ENSG00000160294
MCTS1	ENSG00000232119
MDH2	ENSG00000146701
MDN1	ENSG00000112159
MECP2	ENSG00000169057
MEPCE	ENSG00000146834
METAP2	ENSG00000111142
METTL16	ENSG00000127804
MEX3A	ENSG00000254726
MEX3B	ENSG00000183496
MEX3C	ENSG00000176624
MEX3D	ENSG00000181588

MFAP1	ENSG00000140259
MKI67	ENSG00000148773
MKI67IP	ENSG00000155438
MKRN1	ENSG00000133606
MKRN2	ENSG00000075975
MKRN3	ENSG00000179455
MKRN4P	ENSG00000238222
MOV10	ENSG00000155363
MOV10L1	ENSG00000073146
MPHOSPH10	ENSG00000124383
MRM1	ENSG00000129282
MRPL1	ENSG00000169288
MRPL10	ENSG00000159111
MRPL11	ENSG00000174547
MRPL13	ENSG00000172172
MRPL14	ENSG00000180992
MRPL15	ENSG00000137547
MRPL19	ENSG00000115364
MRPL2	ENSG00000112651
MRPL20	ENSG00000242485
MRPL21	ENSG00000197345
MRPL22	ENSG00000082515
MRPL27	ENSG00000108826
MRPL28	ENSG00000086504
MRPL3	ENSG00000114686
MRPL32	ENSG00000106591
MRPL37	ENSG00000116221
MRPL39	ENSG00000154719
MRPL4	ENSG00000105364
MRPL40	ENSG00000185608
MRPL41	ENSG00000182154
MRPL42	ENSG00000198015
MRPL43	ENSG00000055950
MRPL44	ENSG00000135900
MRPL45	ENSG00000174100
MRPL54	ENSG00000183617
MRPL9	ENSG00000143436
MRPS11	ENSG00000181991
MRPS12	ENSG00000128626
MRPS14	ENSG00000120333
MRPS15	ENSG00000116898
MRPS18C	ENSG00000163319
MRPS21	ENSG00000187145
MRPS23	ENSG00000181610
MRPS24	ENSG00000062582
MRPS26	ENSG00000125901
MRPS27	ENSG00000113048

MRPS28	ENSG00000147586
MRPS30	ENSG00000112996
MRPS31	ENSG00000102738
MRPS35	ENSG00000061794
MRPS5	ENSG00000144029
MRPS7	ENSG00000125445
MRPS9	ENSG00000135972
MRTO4	ENSG00000053372
MSI1	ENSG00000135097
MSI2	ENSG00000153944
MTDH	ENSG00000147649
MTERF	ENSG00000127989
MTHFSD	ENSG00000103248
MTIF2	ENSG00000085760
MTO1	ENSG00000135297
MTPAP	ENSG00000107951
MYBBP1A	ENSG00000132382
MYEF2	ENSG00000104177
MYH9	ENSG00000100345
MYO18A	ENSG00000196535
MYO5A	ENSG00000197535
NAA15	ENSG00000164134
NAA38	ENSG00000128534
NACA2	ENSG00000253506
NAF1	ENSG00000145414
NAP1L1	ENSG00000187109
NAP1L4	ENSG00000205531
NASP	ENSG00000132780
NAT10	ENSG00000135372
NBPF10	ENSG00000163386
NCBP1	ENSG00000136937
NCBP2	ENSG00000114503
NCBP2L	ENSG00000170935
NCL	ENSG00000115053
NCOA5	ENSG00000124160
NDUFV3	ENSG00000160194
NELFE	ENSG00000204356
NFX1	ENSG00000086102
NGDN	ENSG00000129460
NGRN	ENSG00000182768
NHP2	ENSG00000145912
NHP2L1	ENSG00000100138
NIP7	ENSG00000132603
NKAP	ENSG00000101882
NKRF	ENSG00000186416
NLRP11	ENSG00000179873
NMD3	ENSG00000169251

NME1	ENSG00000239672
NMT1	ENSG00000136448
NOA1	ENSG00000084092
NOC2L	ENSG00000188976
NOC3L	ENSG00000173145
NOC4L	ENSG00000184967
NOL10	ENSG00000115761
NOL11	ENSG00000130935
NOL12	ENSG00000256872
NOL6	ENSG00000165271
NOL7	ENSG00000225921
NOL8	ENSG00000198000
NOLC1	ENSG00000166197
NOM1	ENSG00000146909
NOMO1	ENSG00000103512
NOMO2	ENSG00000185164
NOMO3	ENSG00000103226
NONO	ENSG00000147140
NOP14	ENSG00000087269
NOP16	ENSG00000048162
NOP2	ENSG00000111641
NOP56	ENSG00000101361
NOP58	ENSG00000055044
NOP9	ENSG00000196943
NOSIP	ENSG00000142546
NOVA1	ENSG00000139910
NOVA2	ENSG00000104967
NPM1	ENSG00000181163
NPM3	ENSG00000107833
NQO1	ENSG00000181019
NSA2	ENSG00000164346
NSRP1	ENSG00000126653
NSUN2	ENSG00000037474
NSUN5	ENSG00000130305
NSUN6	ENSG00000241058
NTPCR	ENSG00000135778
NUCKS1	ENSG00000069275
NUDT16L1	ENSG00000168101
NUDT21	ENSG00000167005
NUFIP1	ENSG00000083635
NUFIP2	ENSG00000108256
NUPL2	ENSG00000136243
NUSAP1	ENSG00000137804
NVL	ENSG00000143748
NXF1	ENSG00000162231
OASL	ENSG00000135114
OLA1	ENSG00000138430

OTUD4	ENSG00000164164
P4HB	ENSG00000185624
PA2G4	ENSG00000170515
PABPC1	ENSG00000070756
PABPC1L	ENSG00000101104
PABPC1L2A	ENSG00000186288
PABPC1L2B	ENSG00000184388
PABPC3	ENSG00000151846
PABPC4	ENSG00000090621
PABPC4L	ENSG00000254535
PABPC5	ENSG00000174740
PABPN1	ENSG00000100836
PABPN1L	ENSG00000205022
PAK1IP1	ENSG00000111845
PAN3	ENSG00000152520
PAPD5	ENSG00000121274
PARN	ENSG00000140694
PARP1	ENSG00000143799
PARP10	ENSG00000178685
PARP12	ENSG00000059378
PARP14	ENSG00000173193
PATL1	ENSG00000166889
PCBP1	ENSG00000169564
PCBP2	ENSG00000197111
PCBP3	ENSG00000183570
PCBP4	ENSG00000090097
PCDH20	ENSG00000197991
PCDHGA9	ENSG00000261934
PCSK9	ENSG00000169174
PDAP1	ENSG00000106244
PDCD11	ENSG00000148843
PDIA3	ENSG00000167004
PDIA4	ENSG00000155660
PDIA6	ENSG00000143870
PEBP1	ENSG00000089220
PEF1	ENSG00000162517
PEG10	ENSG00000242265
PELP1	ENSG00000141456
PES1	ENSG00000100029
PFN1	ENSG00000108518
PHF5A	ENSG00000100410
PHF6	ENSG00000156531
PIN4	ENSG00000102309
PKM	ENSG00000067225
PKN2	ENSG00000065243
PLEC	ENSG00000178209
PNISR	ENSG00000132424

PNN	ENSG00000100941
PNO1	ENSG00000115946
PNPT1	ENSG00000138035
POLDIP3	ENSG00000100227
POLR1E	ENSG00000137054
POLR2A	ENSG00000181222
POLR2B	ENSG00000047315
POLR2G	ENSG00000168002
POLRMT	ENSG00000099821
POP1	ENSG00000104356
POP7	ENSG00000172336
POU5F1	ENSG00000204531
PPAN	ENSG00000130810
PPAN-P2RY11	ENSG00000243207
PPARGC1A	ENSG00000109819
PPARGC1B	ENSG00000155846
PPHLN1	ENSG00000134283
PPIA	ENSG00000196262
PPIB	ENSG00000166794
PPIE	ENSG00000084072
PPIG	ENSG00000138398
PPIL4	ENSG00000131013
PPP1CC	ENSG00000186298
PPP1R10	ENSG00000204569
PPRC1	ENSG00000148840
PQBP1	ENSG00000102103
PRDX1	ENSG00000117450
PRKDC	ENSG00000253729
PRKRA	ENSG00000180228
PRMT1	ENSG00000126457
PRPF3	ENSG00000117360
PRPF31	ENSG00000105618
PRPF38A	ENSG00000134748
PRPF38B	ENSG00000134186
PRPF40A	ENSG00000196504
PRPF4B	ENSG00000112739
PRPF6	ENSG00000101161
PRPF8	ENSG00000174231
PRR3	ENSG00000204576
PRRC2A	ENSG00000204469
PRRC2A(SUPERCONTIG)	ENSG00000225748
PRRC2B	ENSG00000130723
PRRC2C	ENSG00000117523
PSIP1	ENSG00000164985
PSMC1	ENSG00000100764
PSMD4	ENSG00000159352
PSPC1	ENSG00000121390

PTBP1	ENSG00000011304
PTBP2	ENSG00000117569
PTBP3	ENSG00000119314
PTCD1	ENSG00000106246
PTCD2	ENSG00000049883
PTCD3	ENSG00000132300
PTPN1	ENSG00000196396
PTRF	ENSG00000177469
PTRH1	ENSG00000187024
PUF60	ENSG00000179950
PUM1	ENSG00000134644
PUM2	ENSG00000055917
PURA	ENSG00000185129
PURB	ENSG00000146676
PURG	ENSG00000172733
PUS1	ENSG00000177192
PUS7	ENSG00000091127
PWP1	ENSG00000136045
PWP2	ENSG00000241945
QKI	ENSG00000112531
R3HDM1	ENSG00000048991
R3HDM2	ENSG00000179912
RALY	ENSG00000125970
RALYL	ENSG00000184672
RAN	ENSG00000132341
RARS2	ENSG00000146282
RAVER1	ENSG00000161847
RAVER2	ENSG00000162437
RBBP6	ENSG00000122257
RBFOX1	ENSG00000078328
RBFOX2	ENSG00000100320
RBFOX3	ENSG00000167281
RBM10	ENSG00000182872
RBM11	ENSG00000185272
RBM12	ENSG00000244462
RBM12B	ENSG00000183808
RBM14	ENSG00000239306
RBM15	ENSG00000162775
RBM15B	ENSG00000179837
RBM17	ENSG00000134453
RBM18	ENSG00000119446
RBM19	ENSG00000122965
RBM20	ENSG00000203867
RBM22	ENSG00000086589
RBM23	ENSG00000100461
RBM24	ENSG00000112183
RBM25	ENSG00000119707

RBM26	ENSG00000139746
RBM27	ENSG00000091009
RBM28	ENSG00000106344
RBM3	ENSG00000102317
RBM33	ENSG00000184863
RBM34	ENSG00000188739
RBM38	ENSG00000132819
RBM39	ENSG00000131051
RBM4	ENSG00000173933
RBM41	ENSG00000089682
RBM42	ENSG00000126254
RBM43	ENSG00000184898
RBM44	ENSG00000177483
RBM45	ENSG00000155636
RBM46	ENSG00000151962
RBM47	ENSG00000163694
RBM4B	ENSG00000173914
RBM5	ENSG00000003756
RBM6	ENSG00000004534
RBM7	ENSG00000076053
RBM8A	ENSG00000131795
RBMS1	ENSG00000153250
RBMS2	ENSG00000076067
RBMS2P1	ENSG00000213250
RBMS3	ENSG00000144642
RBMX	ENSG00000147274
RBMX2	ENSG00000134597
RBMXL1	ENSG00000213516
RBMXL2	ENSG00000170748
RBMXL3	ENSG00000175718
RBMY1A1	ENSG00000234414
RBMY1B	ENSG00000242875
RBMY1D	ENSG00000244395
RBMY1E	ENSG00000242389
RBMY1F	ENSG00000169800
RBMY1J	ENSG00000226941
RBPMS	ENSG00000157110
RBPMS2	ENSG00000166831
RC3H1	ENSG00000135870
RC3H2	ENSG00000056586
RCAN2	ENSG00000172348
RCC2	ENSG00000179051
RDM1	ENSG00000187456
RDX	ENSG00000137710
REPIN1	ENSG00000214022
REXO4	ENSG00000148300
RIMS1	ENSG00000079841

RNF113A	ENSG00000125352
RNF113B	ENSG00000139797
RNMTL1	ENSG00000171861
RNPC3	ENSG00000185946
RNPS1	ENSG00000205937
ROCK2	ENSG00000134318
RP11-212D19.4	ENSG00000255663
RP11-603J24.9	ENSG00000257411
RP1-37E16.12	ENSG00000100101
RP9	ENSG00000164610
RPF1	ENSG00000117133
RPF2	ENSG00000197498
RPGR	ENSG00000156313
RPL10	ENSG00000147403
RPL10A	ENSG00000198755
RPL11	ENSG00000142676
RPL12	ENSG00000197958
RPL13	ENSG00000167526
RPL13A	ENSG00000142541
RPL14	ENSG00000188846
RPL15	ENSG00000174748
RPL17	ENSG00000265681
RPL17-C18orf32	ENSG00000215472
RPL18A	ENSG00000105640
RPL19	ENSG00000108298
RPL21	ENSG00000122026
RPL22	ENSG00000116251
RPL22L1	ENSG00000163584
RPL23	ENSG00000125691
RPL23A	ENSG00000198242
RPL24	ENSG00000114391
RPL26	ENSG00000161970
RPL27	ENSG00000131469
RPL27A	ENSG00000166441
RPL28	ENSG00000108107
RPL29	ENSG00000162244
RPL3	ENSG00000100316
RPL30	ENSG00000156482
RPL31	ENSG00000071082
RPL32	ENSG00000144713
RPL35	ENSG00000136942
RPL35A	ENSG00000182899
RPL36	ENSG00000130255
RPL36A	ENSG00000241343
RPL37A	ENSG00000197756
RPL4	ENSG00000174444
RPL5	ENSG00000122406

RPL6	ENSG00000089009
RPL7	ENSG00000147604
RPL7A	ENSG00000148303
RPL7L1	ENSG00000146223
RPL8	ENSG00000161016
RPLP0	ENSG00000089157
RPN1	ENSG00000163902
RPP25	ENSG00000178718
RPP25L	ENSG00000164967
RPP30	ENSG00000148688
RPS10	ENSG00000124614
RPS11	ENSG00000142534
RPS12	ENSG00000112306
RPS13	ENSG00000110700
RPS14	ENSG00000164587
RPS15	ENSG00000115268
RPS15A	ENSG00000134419
RPS16	ENSG00000105193
RPS17	ENSG00000184779
RPS17L	ENSG00000182774
RPS18	ENSG00000231500
RPS18(SUPERCONTIG)	ENSG00000096150
RPS19	ENSG00000105372
RPS19BP1	ENSG00000187051
RPS2	ENSG00000140988
RPS20	ENSG00000008988
RPS21	ENSG00000171858
RPS23	ENSG00000186468
RPS24	ENSG00000138326
RPS25	ENSG00000118181
RPS26	ENSG00000197728
RPS27	ENSG00000177954
RPS27A	ENSG00000143947
RPS27L	ENSG00000185088
RPS28	ENSG00000233927
RPS3	ENSG00000149273
RPS3A	ENSG00000145425
RPS4X	ENSG00000198034
RPS5	ENSG00000083845
RPS6	ENSG00000137154
RPS7	ENSG00000171863
RPS8	ENSG00000142937
RPS9	ENSG00000170889
RPSA	ENSG00000168028
RPUSD2	ENSG00000166133
RPUSD3	ENSG00000156990
RPUSD4	ENSG00000165526

RRBP1	ENSG00000125844
RRP1	ENSG00000160214
RRP12	ENSG00000052749
RRP15	ENSG00000067533
RRP1B	ENSG00000160208
RRP36	ENSG00000124541
RRP7A	ENSG00000189306
RRP8	ENSG00000132275
RRP9	ENSG00000114767
RRS1	ENSG00000179041
RSL1D1	ENSG00000171490
RSRC2	ENSG00000111011
RTCA	ENSG00000137996
RTCB	ENSG00000100220
RTF1	ENSG00000137815
RTN4	ENSG00000115310
S100A16	ENSG00000188643
S100A4	ENSG00000196154
SAFB	ENSG00000160633
SAFB2	ENSG00000130254
SAMD14	ENSG00000167100
SAMD4A	ENSG00000020577
SAMD4B	ENSG00000179134
SAMSN1	ENSG00000155307
SAP18	ENSG00000150459
SARNP	ENSG00000205323
SARS2	ENSG00000104835
SART1	ENSG00000175467
SART3	ENSG00000075856
SBDS	ENSG00000126524
SCAF11	ENSG00000139218
SCAF4	ENSG00000156304
SCAF8	ENSG00000213079
SCG3	ENSG00000104112
SDAD1	ENSG00000198301
SEC23IP	ENSG00000107651
SEC61A1	ENSG00000058262
SEC61B	ENSG00000106803
SEC63	ENSG00000025796
SECISBP2	ENSG00000187742
SECISBP2L	ENSG00000138593
SERBP1	ENSG00000142864
SERPINH1	ENSG00000149257
SETD1A	ENSG00000099381
SETD1B	ENSG00000139718
SF1	ENSG00000168066
SF3A1	ENSG00000099995

SF3A2	ENSG00000104897
SF3A3	ENSG00000183431
SF3B1	ENSG00000115524
SF3B14	ENSG00000115128
SF3B2	ENSG00000087365
SF3B4	ENSG00000143368
SFMBT2	ENSG00000198879
SFN	ENSG00000175793
SFPQ	ENSG00000116560
SFSWAP	ENSG00000061936
SKIV2L2	ENSG00000039123
SLBP	ENSG00000163950
SLC16A3	ENSG00000141526
SLC25A11	ENSG00000108528
SLC25A5	ENSG00000005022
SLC35G6	ENSG00000259224
SLC3A2	ENSG00000168003
SLIRP	ENSG00000119705
SLTM	ENSG00000137776
SMC1A	ENSG00000072501
SMG1	ENSG00000157106
SMNDC1	ENSG00000119953
SND1	ENSG00000197157
SNIP1	ENSG00000163877
SNRNP200	ENSG00000144028
SNRNP35	ENSG00000184209
SNRNP40	ENSG00000060688
SNRNP70	ENSG00000104852
SNRPA	ENSG00000077312
SNRPA1	ENSG00000131876
SNRPB	ENSG00000125835
SNRPB2	ENSG00000125870
SNRPC	ENSG00000124562
SNRPD1	ENSG00000167088
SNRPD2	ENSG00000125743
SNRPD3	ENSG00000100028
SNRPE	ENSG00000182004
SNRPEP3	ENSG00000227624
SNRPF	ENSG00000139343
SNRPG	ENSG00000143977
SNRPN	ENSG00000128739
SNTB2	ENSG00000168807
SNW1	ENSG00000100603
SOGA2	ENSG00000168502
SON	ENSG00000159140
SORBS2	ENSG00000154556
SPATS2	ENSG00000123352

SPATS2L	ENSG00000196141
SPEN	ENSG00000065526
SPTAN1	ENSG00000197694
SPTBN1	ENSG00000115306
SRBD1	ENSG00000068784
SREK1	ENSG00000153914
SRFBP1	ENSG00000151304
SRP14	ENSG00000140319
SRP19	ENSG00000153037
SRP54	ENSG00000100883
SRP68	ENSG00000167881
SRP72	ENSG00000174780
SRPK1	ENSG00000096063
SRPK2	ENSG00000135250
SRPR	ENSG00000182934
SRRM1	ENSG00000133226
SRRM2	ENSG00000167978
SRRT	ENSG00000087087
SRSF1	ENSG00000136450
SRSF10	ENSG00000188529
SRSF10(SUPERCONTIG)	ENSG00000215699
SRSF11	ENSG00000116754
SRSF12	ENSG00000154548
SRSF2	ENSG00000161547
SRSF3	ENSG00000112081
SRSF4	ENSG00000116350
SRSF5	ENSG00000100650
SRSF6	ENSG00000124193
SRSF7	ENSG00000115875
SRSF8	ENSG00000180771
SRSF9	ENSG00000111786
SSB	ENSG00000138385
SSBP1	ENSG00000106028
SSR1	ENSG00000124783
SSRP1	ENSG00000149136
STAU1	ENSG00000124214
STAU2	ENSG00000040341
STIP1	ENSG00000168439
STRAP	ENSG00000023734
STRBP	ENSG00000165209
STXBP1	ENSG00000136854
SUB1	ENSG00000113387
SUCLG1	ENSG00000163541
SUGP1	ENSG00000105705
SUGP2	ENSG00000064607
SUMO1	ENSG00000116030
SUMO2	ENSG00000188612

SUPT16H	ENSG00000092201
SUPT5H	ENSG00000196235
SUPT6H	ENSG00000109111
SUPV3L1	ENSG00000156502
SURF2	ENSG00000148291
SURF6	ENSG00000148296
SYF2	ENSG00000117614
SYNCRIP	ENSG00000135316
SYNE1	ENSG00000131018
SYNJ1	ENSG00000159082
SYNJ2	ENSG00000078269
TACO1	ENSG00000136463
TAF15	ENSG00000172660
TAGLN2	ENSG00000158710
TARDBP	ENSG00000120948
TBCA	ENSG00000171530
TBL2	ENSG00000106638
TBL3	ENSG00000183751
TBRG4	ENSG00000136270
TCEA1	ENSG00000187735
TCERG1	ENSG00000113649
TCF20	ENSG00000100207
TCF25	ENSG00000141002
TCOF1	ENSG00000070814
TCP1	ENSG00000120438
TDRD10	ENSG00000163239
TDRD3	ENSG00000083544
TDRKH	ENSG00000182134
TEFM	ENSG00000172171
TEP1	ENSG00000129566
TES	ENSG00000135269
TEX10	ENSG00000136891
TFAM	ENSG00000108064
TFB1M	ENSG00000029639
TFB2M	ENSG00000162851
TFRC	ENSG00000072274
THRAP3	ENSG00000054118
THUMPD1	ENSG00000066654
THUMPD2	ENSG00000138050
THUMPD3	ENSG00000134077
TIA1	ENSG00000116001
TIAL1	ENSG00000151923
TIPARP	ENSG00000163659
TLR2	ENSG00000137462
TMA16	ENSG00000198498
TMEM63A	ENSG00000196187
TMSB4X	ENSG00000205542

TNPO1	ENSG00000083312
TNRC6A	ENSG00000090905
TNRC6B	ENSG00000100354
TNRC6C	ENSG00000078687
TNS1	ENSG00000079308
TOE1	ENSG00000132773
TOP1	ENSG00000198900
TOP2A	ENSG00000131747
TOP3B	ENSG00000100038
TPD52L2	ENSG00000101150
TPI1	ENSG00000111669
TPR	ENSG00000047410
TPT1	ENSG00000133112
TRA2A	ENSG00000164548
TRA2B	ENSG00000136527
TRAP1	ENSG00000126602
TRIM25	ENSG00000121060
TRIM28	ENSG00000130726
TRIM56	ENSG00000169871
TRIM71	ENSG00000206557
TRIP6	ENSG00000087077
TRMT1	ENSG00000104907
TRMT10A	ENSG00000145331
TRMT10C	ENSG00000174173
TRMT1L	ENSG00000121486
TRMT2A	ENSG00000099899
TRMT44	ENSG00000155275
TRMT6	ENSG00000089195
TRNAU1AP	ENSG00000180098
TROVE2	ENSG00000116747
TRUB2	ENSG00000167112
TSFM	ENSG00000123297
TSNAX	ENSG00000116918
TSR1	ENSG00000167721
TTC14	ENSG00000163728
TUBB	ENSG00000196230
TUBB2A	ENSG00000137267
TUBB2B	ENSG00000137285
TUBB4A	ENSG00000104833
TUFM	ENSG00000178952
TUT1	ENSG00000149016
TWF2	ENSG00000247596
TWISTNB	ENSG00000105849
TXN	ENSG00000136810
U2AF1	ENSG00000160201
U2AF1L4	ENSG00000161265
U2AF2	ENSG00000063244

U2SURP	ENSG00000163714
UBA1	ENSG00000130985
UBAP2	ENSG00000137073
UBAP2L	ENSG00000143569
UBC	ENSG00000150991
UBE2D1	ENSG00000072401
UBE2D2	ENSG00000131508
UBE2D3	ENSG00000109332
UBE2I	ENSG00000103275
UBE2L3	ENSG00000185651
UBE2N	ENSG00000177889
UBE2O	ENSG00000175931
UBFD1	ENSG00000103353
UBTF	ENSG00000108312
UCHL5	ENSG00000116750
UHMK1	ENSG00000152332
UNK	ENSG00000132478
UPF1	ENSG00000005007
UPF3B	ENSG00000125351
URB1	ENSG00000142207
URB2	ENSG00000135763
USO1	ENSG00000138768
USP10	ENSG00000103194
USP36	ENSG00000055483
UTP11L	ENSG00000183520
UTP14A	ENSG00000156697
UTP14C	ENSG00000253797
UTP15	ENSG00000164338
UTP18	ENSG00000011260
UTP20	ENSG00000120800
UTP23	ENSG00000147679
UTP3	ENSG00000132467
UTP6	ENSG00000108651
VCP	ENSG00000165280
WBP11	ENSG00000084463
WBSCR16	ENSG00000174374
WBSCR22	ENSG00000071462
WDR3	ENSG00000065183
WDR33	ENSG00000136709
WDR36	ENSG00000134987
WDR43	ENSG00000163811
WDR46	ENSG00000227057
WDR46(SUPERCONTIG)	ENSG00000236222
WDR6	ENSG00000178252
WDR75	ENSG00000115368
WIBG	ENSG00000170473
XIRP1	ENSG00000168334

XPO1	ENSG00000082898
XPO5	ENSG00000124571
XRCC5	ENSG00000079246
XRCC6	ENSG00000196419
XRN1	ENSG00000114127
XRN2	ENSG00000088930
YARS	ENSG00000134684
YARS2	ENSG00000139131
YBX1	ENSG00000065978
YBX2	ENSG00000006047
YBX3	ENSG00000060138
YLPM1	ENSG00000119596
YTHDC1	ENSG00000083896
YTHDC2	ENSG00000047188
YTHDF1	ENSG00000149658
YTHDF2	ENSG00000198492
YTHDF3	ENSG00000185728
YWHAE	ENSG00000108953
YWHAG	ENSG00000170027
YWHAH	ENSG00000128245
YWHAQ	ENSG00000134308
YWHAZ	ENSG00000164924
ZAK	ENSG00000091436
ZBTB11	ENSG00000066422
ZC3H10	ENSG00000135482
ZC3H11A	ENSG00000058673
ZC3H12A	ENSG00000163874
ZC3H12B	ENSG00000102053
ZC3H12C	ENSG00000149289
ZC3H12D	ENSG00000178199
ZC3H13	ENSG00000123200
ZC3H14	ENSG00000100722
ZC3H15	ENSG00000065548
ZC3H18	ENSG00000158545
ZC3H3	ENSG00000014164
ZC3H4	ENSG00000130749
ZC3H6	ENSG00000188177
ZC3H7A	ENSG00000122299
ZC3H7B	ENSG00000100403
ZC3H8	ENSG00000144161
ZC3HAV1	ENSG00000105939
ZC3HAV1L	ENSG00000146858
ZCCHC11	ENSG00000134744
ZCCHC17	ENSG00000121766
ZCCHC24	ENSG00000165424
ZCCHC3	ENSG00000177764
ZCCHC6	ENSG00000083223

ZCCHC7	ENSG00000147905
ZCCHC8	ENSG00000033030
ZCCHC9	ENSG00000131732
ZCRB1	ENSG00000139168
ZFC3H1	ENSG00000133858
ZFP36	ENSG00000128016
ZFP36L1	ENSG00000185650
ZFP36L2	ENSG00000152518
ZFR	ENSG00000056097
ZFR2	ENSG00000105278
ZGPAT	ENSG00000197114
ZMAT3	ENSG00000172667
ZMAT5	ENSG00000100319
ZNF106	ENSG00000103994
ZNF207	ENSG00000010244
ZNF239	ENSG00000196793
ZNF326	ENSG00000162664
ZNF346	ENSG00000113761
ZNF385A	ENSG00000161642
ZNF579	ENSG00000218891
ZNF593	ENSG00000142684
ZNF598	ENSG00000167962
ZNF622	ENSG00000173545
ZNF638	ENSG00000075292
ZNF74	ENSG00000185252
ZNF768	ENSG00000169957
ZNFX1	ENSG00000124201
ZNHIT6	ENSG00000117174
ZRANB2	ENSG00000132485
ZRSR1	ENSG00000212643
ZRSR2	ENSG00000169249
ZYX	ENSG00000159840

Supplemental Table S2	
Census database	
Gene Name	Gene ID
RPS11	ENSG00000142534
ERAL1	ENSG00000132591
DDX27	ENSG00000124228
DEK	ENSG00000124795
PSMA6	ENSG00000100902
TRIM56	ENSG00000169871
TRIM71	ENSG00000206557
UPF2	ENSG00000151461
FARS2	ENSG00000145982
FDXACB1	ENSG00000262028
ALKBH8	ENSG00000137760
ZNF579	ENSG00000218891
POLR2B	ENSG00000047315
WDR12	ENSG00000138442
RPF1	ENSG00000117133
MRPS34	ENSG00000074071
UBTF	ENSG00000108312
CDC5L	ENSG00000096401
DAZAP1	ENSG00000071626
MSI1	ENSG00000135097
MSI2	ENSG00000153944
TARDBP	ENSG00000120948
TRIT1	ENSG00000043514
MRPL36	ENSG00000171421
KPNB1	ENSG00000108424
ZNFX1	ENSG00000124201
SNRNP27	ENSG00000124380
RBMS2	ENSG00000076067
RBMS1	ENSG00000153250
RBMS3	ENSG00000144642
LARS2	ENSG00000011376
KIAA0020	ENSG00000080608
RBFOX1	ENSG00000078328
RBFOX2	ENSG00000100320
RBFOX3	ENSG00000267483
CWF19L1	ENSG00000095485
CWF19L2	ENSG00000152404
ZC3H15	ENSG00000065548
LONP1	ENSG00000196365
DBR1	ENSG00000138231
RRP15	ENSG00000067533
L1TD1	ENSG00000240563
NMD3	ENSG00000169251

SRSF10	ENSG00000215699
SRSF12	ENSG00000154548
SRSF2	ENSG00000161547
SRSF8	ENSG00000180771
RSL1D1	ENSG00000171490
DAP3	ENSG00000132676
NARS2	ENSG00000137513
RBM25	ENSG00000119707
POLR2E	ENSG00000099817
TOE1	ENSG00000132773
TSFM	ENSG00000123297
TPR	ENSG00000047410
STAU1	ENSG00000124214
STAU2	ENSG00000040341
TRMT1L	ENSG00000121486
METTL3	ENSG00000165819
REXO2	ENSG00000076043
MRPL52	ENSG00000172590
WDR3	ENSG00000065183
CNOT11	ENSG00000158435
YTHDC1	ENSG00000257413
METTL2A	ENSG00000087995
METTL2B	ENSG00000165055
ZNF326	ENSG00000162664
AKAP8	ENSG00000105127
AKAP8L	ENSG00000011243
RPS14	ENSG00000164587
ELAVL1	ENSG00000066044
ELAVL2	ENSG00000107105
ELAVL3	ENSG00000196361
ELAVL4	ENSG00000162374
PNN	ENSG00000100941
DDX41	ENSG00000183258
TNPO2	ENSG00000105576
TNPO1	ENSG00000083312
PUSL1	ENSG00000169972
RBM43	ENSG00000184898
ZGPAT	ENSG00000197114
SCAF11	ENSG00000139218
USP39	ENSG00000168883
RPL38	ENSG00000172809
NARS	ENSG00000134440
RPS16	ENSG00000105193
CLASRP	ENSG00000104859
RPS9	ENSG00000170889
TOP3B	ENSG00000100038
NCBP2L	ENSG00000170935

NCBP2	ENSG00000114503
SMG9	ENSG00000105771
FTSJ2	ENSG00000122687
DDX56	ENSG00000136271
RBM28	ENSG00000106344
UTP11L	ENSG00000183520
MRPS18B	ENSG00000223775
RPL14	ENSG00000188846
RPL37	ENSG00000145592
GEMIN5	ENSG00000082516
MKRN1	ENSG00000133606
MKRN2	ENSG00000075975
MKRN3	ENSG00000179455
NOA1	ENSG00000084092
PINX1	ENSG00000254093
HRSP12	ENSG00000132541
TDRD9	ENSG00000156414
DHX30	ENSG00000132153
DHX29	ENSG00000067248
DHX57	ENSG00000163214
YTHDC2	ENSG00000047188
DHX36	ENSG00000174953
DHX9	ENSG00000135829
EIF3G	ENSG00000130811
NOL10	ENSG00000115761
UTP15	ENSG00000164338
GATC	ENSG00000257218
TCOF1	ENSG00000070814
NONO	ENSG00000147140
PSPC1	ENSG00000121390
SFPQ	ENSG00000116560
NUDT21	ENSG00000167005
MRPL19	ENSG00000115364
URM1	ENSG00000167118
MRPL53	ENSG00000204822
RBM48	ENSG00000127993
REPIN1	ENSG00000214022
ZC3H12B	ENSG00000102053
ZC3H12C	ENSG00000149289
ZC3H12D	ENSG00000178199
ZC3H12A	ENSG00000163874
TRMT5	ENSG00000126814
TRMT61A	ENSG00000166166
PABPN1L	ENSG00000205022
PABPN1	ENSG00000100836
LSG1	ENSG00000041802
GNL1	ENSG00000206412

EEF1G	ENSG00000254772
RBM33	ENSG00000184863
RPP25	ENSG00000178718
RPP25L	ENSG00000164967
RPP38	ENSG00000152464
GARS	ENSG00000106105
DRG2	ENSG00000108591
TRMU	ENSG00000100416
GNL2	ENSG00000134697
CPSF2	ENSG00000165934
CD3EAP	ENSG00000117877
HDLBP	ENSG00000115677
RDM1	ENSG00000187456
BYSL	ENSG00000112578
GEMIN8	ENSG00000046647
XPO5	ENSG00000124571
ZNF622	ENSG00000173545
DZIP1	ENSG00000134874
DZIP1L	ENSG00000158163
GSPT1	ENSG00000103342
HBS1L	ENSG00000112339
GSPT2	ENSG00000268273
EIF2D	ENSG00000143486
METTL1	ENSG00000037897
RNASEH1	ENSG00000171865
ZNHIT6	ENSG00000117174
DARS2	ENSG00000117593
SNRPD2	ENSG00000125743
DICER1	ENSG00000100697
DYNLL1	ENSG00000088986
ZNF638	ENSG00000075292
SAMD4B	ENSG00000179134
SAMD4A	ENSG00000020577
RTF1	ENSG00000137815
CNOT4	ENSG00000080802
TARBP2	ENSG00000139546
PRKRA	ENSG00000180228
GLE1	ENSG00000119392
EXOSC2	ENSG00000130713
SSU72	ENSG00000160075
GTPBP10	ENSG00000105793
RPL10A	ENSG00000198755
HENMT1	ENSG00000162639
THOC2	ENSG00000125676
ENDOG	ENSG00000167136
EXOG	ENSG00000157036
ASH1L	ENSG00000116539

TRMT10C	ENSG00000174173
TRMT10A	ENSG00000145331
TRMT10B	ENSG00000165275
MRPL33	ENSG00000243147
SSB	ENSG00000138385
LARP7	ENSG00000174720
MRPL24	ENSG00000143314
RNASEK	ENSG00000219200
PELO	ENSG00000152684
QARS	ENSG00000172053
IPO4	ENSG00000196497
CAPRIN1	ENSG00000135387
CAPRIN2	ENSG00000110888
PNLDC1	ENSG00000146453
PARN	ENSG00000140694
TIAL1	ENSG00000151923
TIA1	ENSG00000116001
CDC40	ENSG00000168438
ZNF598	ENSG00000167962
RCL1	ENSG00000120158
RTCA	ENSG00000137996
MRPL38	ENSG00000204316
ADAT2	ENSG00000189007
BOP1	ENSG00000261236
U2SURP	ENSG00000163714
DUS3L	ENSG00000141994
CSTF3	ENSG00000176102
SRSF11	ENSG00000116754
SREK1	ENSG00000153914
POLR1E	ENSG00000137054
EXOSC7	ENSG00000075914
EXOSC8	ENSG00000120699
EXOSC9	ENSG00000123737
MRPL12	ENSG00000262814
RPS28	ENSG00000233927
TEFM	ENSG00000172171
RPL12	ENSG00000197958
ZC3H3	ENSG00000014164
EIF6	ENSG00000242372
NELFE	ENSG00000206268
EIF2B3	ENSG00000070785
APOBEC2	ENSG00000124701
APOBEC3H	ENSG00000100298
APOBEC3F	ENSG00000128394
APOBEC3G	ENSG00000239713
APOBEC1	ENSG00000111701
ZC3H7A	ENSG00000122299

ZC3H7B	ENSG00000100403
NOP9	ENSG00000196943
PQBP1	ENSG00000102103
PATL2	ENSG00000229474
PATL1	ENSG00000166889
EDC3	ENSG00000179151
WBP4	ENSG00000120688
GLTSCR2	ENSG00000105373
RPS18	ENSG00000226225
ASCC1	ENSG00000138303
EIF5A	ENSG00000132507
EIF5A2	ENSG00000163577
EIF5AL1	ENSG00000253626
MRPL47	ENSG00000136522
BOLL	ENSG00000152430
DAZL	ENSG00000092345
DAZ1	ENSG00000188120
DAZ2	ENSG00000205944
DAZ3	ENSG00000187191
DAZ4	ENSG00000205916
INTS1	ENSG00000164880
RPLP1	ENSG00000137818
MRPS11	ENSG00000181991
WDR43	ENSG00000163811
TRMT112	ENSG00000173113
PPP1R8	ENSG00000117751
FBXO17	ENSG00000104835
MTHFSD	ENSG00000103248
SON	ENSG00000159140
SPATS2L	ENSG00000196141
SPATS2	ENSG00000123352
MAEL	ENSG00000143194
RRP1B	ENSG00000160208
RRP1	ENSG00000160214
ZC3HAV1	ENSG00000105939
PARP12	ENSG00000059378
TIPARP	ENSG00000163659
MRPS17	ENSG00000239789
U2AF2	ENSG00000063244
UHMK1	ENSG00000152332
NUP153	ENSG00000124789
CSDE1	ENSG00000009307
POLR2L	ENSG00000177700
NKRF	ENSG00000186416
MRPL48	ENSG00000175581
XRCC6	ENSG00000196419
SUPT5H	ENSG00000196235

TSR3	ENSG00000007520
XRN2	ENSG00000088930
RSRC1	ENSG00000174891
TDRD5	ENSG00000162782
POLR2J	ENSG00000005075
POLR2J2	ENSG00000228049
POLR2J3	ENSG00000168255
SAFB2	ENSG00000130254
SAFB	ENSG00000160633
SLTM	ENSG00000137776
BRIX1	ENSG00000113460
POLR2I	ENSG00000105258
PWP2	ENSG00000241945
ELAC2	ENSG00000006744
ZCCHC14	ENSG00000140948
ZCCHC2	ENSG00000141664
LARS	ENSG00000133706
MRPL28	ENSG00000086504
CNOT1	ENSG00000125107
DDX18	ENSG00000088205
TSEN15	ENSG00000198860
USP10	ENSG00000103194
SMNDC1	ENSG00000119953
THUMPD2	ENSG00000138050
THUMPD3	ENSG00000134077
SND1	ENSG00000197157
LSM4	ENSG00000130520
EIF3M	ENSG00000149100
MRPL34	ENSG00000130312
SRP72	ENSG00000174780
SUGP1	ENSG00000105705
SUGP2	ENSG00000064607
RPS3	ENSG00000149273
RPS27A	ENSG00000143947
CCAR2	ENSG00000158941
CCAR1	ENSG00000060339
DDX49	ENSG00000105671
MRPL4	ENSG00000105364
SFSWAP	ENSG00000061936
MRPS12	ENSG00000128626
EIF1	ENSG00000173812
EIF1B	ENSG00000114784
CLP1	ENSG00000172409
ZMAT5	ENSG00000100319
SF3B3	ENSG00000189091
PLD6	ENSG00000179598
QTRTD1	ENSG00000151576

QTRT1	ENSG00000213339
PIH1D1	ENSG00000104872
SWT1	ENSG00000116668
EXOSC4	ENSG00000178896
EXOSC6	ENSG00000223496
PTRH1	ENSG00000187024
INTS8	ENSG00000164941
EXOSC10	ENSG00000171824
CASC3	ENSG00000108349
GTF3A	ENSG00000122034
MRPL54	ENSG00000183617
GTPBP3	ENSG00000130299
UBA1	ENSG00000130985
LSM12	ENSG00000161654
HINT3	ENSG00000111911
CCDC59	ENSG00000133773
POLDIP3	ENSG00000100227
RPL32	ENSG00000144713
AZGP1	ENSG00000160862
EMG1	ENSG00000268439
C17orf85	ENSG00000074356
N4BP1	ENSG00000102921
NYNRIN	ENSG00000205978
KHNYN	ENSG00000100441
NOP2	ENSG00000111641
NSUN4	ENSG00000117481
NSUN3	ENSG00000178694
NSUN6	ENSG00000241058
PAN2	ENSG00000135473
ZCCHC9	ENSG00000131732
RRP9	ENSG00000114767
TNRC6A	ENSG00000090905
TNRC6B	ENSG00000100354
TNRC6C	ENSG00000078687
RP9	ENSG00000164610
MRPL15	ENSG00000137547
NGDN	ENSG00000129460
LSM10	ENSG00000181817
PUS1	ENSG00000177192
MAK16	ENSG00000198042
RPS13	ENSG00000110700
SNRNP200	ENSG00000144028
PRDX1	ENSG00000117450
TSN	ENSG00000211460
NUFIP2	ENSG00000108256
RBM19	ENSG00000122965
TDRKH	ENSG00000182134

NOP58	ENSG00000055044
RBM6	ENSG00000004534
RBM10	ENSG00000182872
RBM5	ENSG00000003756
RBM44	ENSG00000177483
OASL	ENSG00000135114
OAS1	ENSG00000089127
OAS2	ENSG00000111335
OAS3	ENSG00000111331
SF3A3	ENSG00000183431
NOC3L	ENSG00000173145
MYEF2	ENSG00000104177
HNRNPM	ENSG00000099783
C11orf68	ENSG00000175573
MRPL10	ENSG00000159111
RNASE10	ENSG00000182545
CALR	ENSG00000179218
CANX	ENSG00000127022
CALR3	ENSG00000269058
RPP14	ENSG00000163684
RPS8	ENSG00000142937
PDCD7	ENSG00000090470
RPS19BP1	ENSG00000187051
TRMT6	ENSG00000089195
RPL4	ENSG00000174444
PRPF3	ENSG00000265228
RARS	ENSG00000113643
RARS2	ENSG00000146282
APOBEC4	ENSG00000173627
ZCCHC24	ENSG00000165424
RPS21	ENSG00000171858
DDX24	ENSG00000089737
RTCB	ENSG00000100220
MBNL2	ENSG00000139793
MBNL3	ENSG00000076770
MBNL1	ENSG00000152601
ALYREF	ENSG00000183684
LSMD1	ENSG00000183011
PUS7	ENSG00000091127
PUS7L	ENSG00000129317
MRPS24	ENSG00000062582
RBBP6	ENSG00000122257
RPL19	ENSG00000108298
NOL3	ENSG00000140939
RPS27	ENSG00000177954
RPS27L	ENSG00000185088
PTRF	ENSG00000177469

MRPS18A	ENSG00000096080
SLBP	ENSG00000163950
EARS2	ENSG00000103356
SMAD1	ENSG00000170365
SMAD2	ENSG00000175387
SMAD5	ENSG00000113658
SMAD9	ENSG00000120693
SMAD3	ENSG00000166949
SMAD4	ENSG00000141646
SMAD7	ENSG00000101665
SMAD6	ENSG00000137834
PRPF39	ENSG00000185246
SRP68	ENSG00000167881
ADARB2	ENSG00000185736
ADAD1	ENSG00000164113
ADAD2	ENSG00000140955
ADAT1	ENSG00000065457
ADARB1	ENSG00000197381
ADAR	ENSG00000160710
CLK3	ENSG00000179335
CLK4	ENSG00000113240
PRPF4B	ENSG00000112739
CLK1	ENSG00000013441
CLK2	ENSG00000176444
LSM3	ENSG00000170860
FAM46A	ENSG00000112773
CRYZ	ENSG00000116791
KIN	ENSG00000151657
RPS2	ENSG00000140988
DUS4L	ENSG00000105865
EIF4A1	ENSG00000161960
EIF4A2	ENSG00000156976
EIF4A3	ENSG00000141543
IGF2BP1	ENSG00000159217
IGF2BP2	ENSG00000073792
IGF2BP3	ENSG00000136231
UBAP2	ENSG00000137073
UBAP2L	ENSG00000143569
SNRPE	ENSG00000182004
EIF2B5	ENSG00000145191
CRNKL1	ENSG00000101343
POLRMT	ENSG00000099821
METTL10	ENSG00000203791
MRPL41	ENSG00000182154
MRPS7	ENSG00000125445
NSUN5	ENSG00000130305
NSUN7	ENSG00000179299

MTG1	ENSG00000254536
MRPS28	ENSG00000147586
TDRD10	ENSG00000163239
RPUSD1	ENSG00000007376
NHP2	ENSG00000145912
XAB2	ENSG00000076924
FAU	ENSG00000149806
RRP36	ENSG00000124541
GRWD1	ENSG00000105447
ENDOV	ENSG00000173818
VARSL	ENSG00000206476
MRRF	ENSG00000148187
MTFMT	ENSG00000103707
C12orf65	ENSG00000130921
MRTO4	ENSG00000053372
RBM26	ENSG00000139746
RBM27	ENSG00000091009
DIS3	ENSG00000083520
DIS3L	ENSG00000166938
DIS3L2	ENSG00000144535
C1QBP	ENSG00000108561
SUB1	ENSG00000113387
MRPS33	ENSG00000090263
PRPF6	ENSG00000101161
SMG8	ENSG00000167447
PSTK	ENSG00000179988
GAPDH	ENSG00000111640
TNPO3	ENSG00000064419
IPO13	ENSG00000117408
MOV10	ENSG00000155363
MOV10L1	ENSG00000073146
PARP1	ENSG00000143799
MRPS35	ENSG00000061794
GPKOW	ENSG00000268675
SETD1A	ENSG00000099381
SETD1B	ENSG00000139718
SF3A2	ENSG00000104897
AFF1	ENSG00000172493
AFF3	ENSG00000144218
AFF4	ENSG00000072364
AFF2	ENSG00000269754
PARK7	ENSG00000116288
TRNAU1AP	ENSG00000180098
SRBD1	ENSG00000068784
EIF3D	ENSG00000100353
NOP10	ENSG00000182117
WARS	ENSG00000140105

MATR3	ENSG00000015479
SNRPD1	ENSG00000167088
SNRPC	ENSG00000124562
FXR1	ENSG00000114416
FXR2	ENSG00000129245
FMR1	ENSG00000102081
MEPCE	ENSG00000146834
KAT8	ENSG00000103510
CIRBP	ENSG00000099622
RBM3	ENSG00000102317
CNOT7	ENSG00000198791
CNOT8	ENSG00000155508
A1CF	ENSG00000148584
RBM46	ENSG00000151962
RBM47	ENSG00000163694
DND1	ENSG00000256453
HNRNPR	ENSG00000125944
SYNCRIP	ENSG00000135316
ABCE1	ENSG00000164163
GPATCH4	ENSG00000160818
PAIP1	ENSG00000172239
RBM20	ENSG00000203867
GNL3	ENSG00000163938
GNL3L	ENSG00000130119
FAM120A	ENSG00000048828
FAM120B	ENSG00000112584
FAM120C	ENSG00000184083
KHDC1	ENSG00000135314
KHDC1L	ENSG00000243501
RPL10L	ENSG00000165496
RPL10	ENSG00000147403
EIF2S1	ENSG00000134001
MRPL20	ENSG00000242485
EPRS	ENSG00000136628
DHX58	ENSG00000260178
DDX58	ENSG00000107201
IFIH1	ENSG00000115267
CDK5RAP1	ENSG00000101391
HABP4	ENSG00000130956
SERBP1	ENSG00000142864
METTL5	ENSG00000138382
ZCCHC7	ENSG00000147905
WDR36	ENSG00000134987
CCDC86	ENSG00000110104
C2orf15	ENSG00000241962
MRPL30	ENSG00000185414
RPS26	ENSG00000197728

TEX13A	ENSG00000133149
BARD1	ENSG00000138376
XPO4	ENSG00000132953
RUVBL1	ENSG00000175792
IMP4	ENSG00000136718
DDX52	ENSG00000141141
THOC1	ENSG00000079134
TRPT1	ENSG00000149743
NHP2L1	ENSG00000100138
EIF2B2	ENSG00000119718
RNF113B	ENSG00000139797
RNF113A	ENSG00000125352
INTS10	ENSG00000104613
LENG9	ENSG00000262887
RPS29	ENSG00000213741
NOP16	ENSG00000048162
U2AF1	ENSG00000160201
U2AF1L4	ENSG00000161265
RANBP6	ENSG00000137040
IPO5	ENSG00000065150
THOC7	ENSG00000163634
DNMT3B	ENSG00000088305
GFM1	ENSG00000168827
RNASE9	ENSG00000188655
PLRG1	ENSG00000171566
REXO1	ENSG00000079313
ZC3HAV1L	ENSG00000146858
LSM14A	ENSG00000262860
LSM14B	ENSG00000149657
LUZP4	ENSG00000267900
TDRD6	ENSG00000180113
TDRD15	ENSG00000218819
MRPS22	ENSG00000175110
EEF1B2	ENSG00000114942
EEF1D	ENSG00000104529
SIDT1	ENSG00000072858
SIDT2	ENSG00000149577
TRMT44	ENSG00000155275
GPATCH8	ENSG00000186566
WDR4	ENSG00000160193
SLIRP	ENSG00000119705
ATXN1	ENSG00000124788
ATXN1L	ENSG00000224470
YTHDF1	ENSG00000149658
YTHDF2	ENSG00000198492
YTHDF3	ENSG00000185728
LRPPRC	ENSG00000138095

TFIP11	ENSG00000100109
ILF2	ENSG00000143621
STRBP	ENSG00000165209
ILF3	ENSG00000129351
TCERG1	ENSG00000113649
NXF5	ENSG00000126952
NXF2B	ENSG00000185945
NXF2	ENSG00000269437
NXF1	ENSG00000162231
NXF3	ENSG00000147206
ZRSR1	ENSG00000212643
ZRSR2	ENSG00000169249
MRPL35	ENSG00000132313
RRS1	ENSG00000179041
PRKDC	ENSG00000253729
NOC2L	ENSG00000188976
MTIF3	ENSG00000122033
NOL11	ENSG00000130935
EXOSC3	ENSG00000107371
PNPT1	ENSG00000138035
TSNAX	ENSG00000116918
RIOK1	ENSG00000124784
RIOK3	ENSG00000101782
PIWIL1	ENSG00000125207
PIWIL2	ENSG00000197181
PIWIL3	ENSG00000184571
PIWIL4	ENSG00000134627
PEG10	ENSG00000242265
ZCCHC5	ENSG00000179300
POP7	ENSG00000172336
FASTKD3	ENSG00000124279
TBRG4	ENSG00000136270
FASTK	ENSG00000164896
TRMT13	ENSG00000122435
IREB2	ENSG00000136381
ACO1	ENSG00000122729
ZCCHC3	ENSG00000177764
SNUPN	ENSG00000169371
PTRHD1	ENSG00000184924
HARS	ENSG00000170445
HARS2	ENSG00000112855
HNRNPK	ENSG00000165119
PCBP1	ENSG00000169564
PCBP2	ENSG00000197111
PCBP3	ENSG00000183570
PCBP4	ENSG00000090097
AQR	ENSG00000021776

ICT1	ENSG00000167862
UNK	ENSG00000132478
UNKL	ENSG00000059145
CEBPZ	ENSG00000115816
MRPS9	ENSG00000135972
THG1L	ENSG00000113272
ZCCHC13	ENSG00000187969
CNBP	ENSG00000169714
SRRT	ENSG00000087087
CD2BP2	ENSG00000169217
SNRPG	ENSG00000143977
APTX	ENSG00000137074
RPL17	ENSG00000265681
BMS1	ENSG00000165733
SARS	ENSG00000031698
SARS2	ENSG00000269547
PAIP2	ENSG00000120727
PAIP2B	ENSG00000124374
GEMIN4	ENSG00000179409
PNRC2	ENSG00000189266
FASTKD2	ENSG00000118246
GEMIN6	ENSG00000152147
DDX20	ENSG00000064703
DDX39A	ENSG00000123136
DDX39B	ENSG00000237889
DHX37	ENSG00000150990
ZNF346	ENSG00000113761
MRPL50	ENSG00000136897
RNASE1	ENSG00000129538
RNASE2	ENSG00000169385
RNASE3	ENSG00000169397
RNASE4	ENSG00000258818
RNASE6	ENSG00000169413
RNASE7	ENSG00000165799
RNASE8	ENSG00000173431
ANG	ENSG00000214274
DYNC1H1	ENSG00000197102
FTSJ3	ENSG00000108592
SF3B2	ENSG00000087365
CTU2	ENSG00000174177
XPOT	ENSG00000184575
RBMXL1	ENSG00000213516
RBMXL2	ENSG00000170748
RBMXL3	ENSG00000175718
RBMY1A1	ENSG00000234414
RBMY1B	ENSG00000242875
RBMY1D	ENSG00000244395

RBMY1E	ENSG00000242389
RBMY1F	ENSG00000169800
RBMY1J	ENSG00000226941
RBMX	ENSG00000147274
PPP1R10	ENSG00000235291
PRR3	ENSG00000206491
DKC1	ENSG00000130826
MRPL17	ENSG00000158042
TRA2A	ENSG00000164548
TRA2B	ENSG00000136527
RPL26L1	ENSG00000037241
RPL26	ENSG00000161970
AUH	ENSG00000148090
EIF3C	ENSG00000184110
EIF3CL	ENSG00000205609
SCAF8	ENSG00000213079
SCAF4	ENSG00000156304
EEF2K	ENSG00000103319
RPL6	ENSG00000089009
MRPL11	ENSG00000174547
G3BP1	ENSG00000145907
G3BP2	ENSG00000138757
MRPS26	ENSG00000125901
SF3B5	ENSG00000169976
DHX34	ENSG00000134815
UTP23	ENSG00000147679
SNRNP48	ENSG00000168566
PRPF38B	ENSG00000134186
HEXIM1	ENSG00000186834
HEXIM2	ENSG00000168517
SRRM2	ENSG00000167978
SRRM3	ENSG00000262321
PIH1D3	ENSG00000080572
DHX40	ENSG00000108406
DHX38	ENSG00000140829
TRMT61B	ENSG00000171103
CSDC2	ENSG00000172346
CARHSP1	ENSG00000153048
POP4	ENSG00000105171
LARP6	ENSG00000166173
RNPC3	ENSG00000185946
RPL30	ENSG00000156482
RUVBL2	ENSG00000183207
DRG1	ENSG00000185721
TRIM25	ENSG00000121060
RQCD1	ENSG00000144580
MRPS18C	ENSG00000163319

MRPL45	ENSG00000174100
RPLP2	ENSG00000177600
SRP19	ENSG00000153037
RPS24	ENSG00000138326
AKAP17A	ENSG00000197976
NXT2	ENSG00000101888
NXT1	ENSG00000132661
MRPL9	ENSG00000143436
RPL27	ENSG00000131469
RPL24	ENSG00000114391
CNOT10	ENSG00000182973
ABT1	ENSG00000146109
DDX25	ENSG00000109832
DDX19A	ENSG00000168872
DDX19B	ENSG00000260537
MRPS30	ENSG00000112996
SMG1	ENSG00000157106
EXOSC5	ENSG00000077348
AIMP2	ENSG00000106305
BZW2	ENSG00000136261
BZW1	ENSG00000082153
PARS2	ENSG00000162396
INTS12	ENSG00000138785
EIF3H	ENSG00000147677
SKIV2L	ENSG00000223493
RC3H2	ENSG00000056586
RC3H1	ENSG00000135870
ZFP36	ENSG00000128016
ZFP36L1	ENSG00000185650
ZFP36L2	ENSG00000152518
ANKHD1	ENSG00000131503
ANKRD17	ENSG00000132466
HELZ2	ENSG00000130589
UPF1	ENSG00000005007
DROSHA	ENSG00000113360
MRPL37	ENSG00000116221
PUS10	ENSG00000162927
NUDT16	ENSG00000198585
NUDT16L1	ENSG00000168101
TRMT11	ENSG00000066651
PET112	ENSG00000059691
NUFIP1	ENSG00000083635
PURA	ENSG00000185129
PURB	ENSG00000146676
PURG	ENSG00000172733
ZRANB2	ENSG00000132485
THOC5	ENSG00000100296

TSEN54	ENSG00000182173
SRP9	ENSG00000143742
PIN4	ENSG00000102309
ZC3H14	ENSG00000100722
CNOT2	ENSG00000111596
TARBP1	ENSG00000059588
AGO1	ENSG00000092847
AGO2	ENSG00000123908
AGO3	ENSG00000126070
AGO4	ENSG00000134698
LARP4B	ENSG00000107929
LARP4	ENSG00000161813
SNW1	ENSG00000100603
EZH2	ENSG00000106462
EIF3B	ENSG00000106263
RPP30	ENSG00000148688
ZMAT3	ENSG00000172667
PTGES3	ENSG00000110958
KARS	ENSG00000065427
RAN	ENSG00000132341
SNRPA	ENSG00000077312
SNRPB2	ENSG00000125870
HEATR1	ENSG00000119285
PTGES3L-AARSD1	ENSG00000108825
AARSD1	ENSG00000266967
CPSF3	ENSG00000119203
CPSF3L	ENSG00000127054
CWC25	ENSG00000108296
RPS5	ENSG00000083845
POLR2D	ENSG00000144231
C9orf114	ENSG00000198917
MRPL44	ENSG00000135900
ETF1	ENSG00000120705
GAR1	ENSG00000109534
MVP	ENSG00000013364
RPF2	ENSG00000197498
ZC3H18	ENSG00000158545
INTS5	ENSG00000185085
INTS3	ENSG00000143624
LSM6	ENSG00000164167
SNRPF	ENSG00000139343
TUT1	ENSG00000149016
MTPAP	ENSG00000107951
EIF2AK1	ENSG00000086232
EIF2AK2	ENSG00000055332
EIF2AK3	ENSG00000172071
EIF2AK4	ENSG00000128829

MRPL22	ENSG00000082515
PES1	ENSG00000100029
LSM7	ENSG00000130332
PAPD4	ENSG00000164329
ZCCHC11	ENSG00000134744
ZCCHC6	ENSG00000083223
KHDRBS1	ENSG00000121774
KHDRBS2	ENSG00000112232
KHDRBS3	ENSG00000131773
QKI	ENSG00000112531
SF1	ENSG00000168066
LRRC47	ENSG00000130764
ISY1	ENSG00000240682
SRRM1	ENSG00000133226
IPO9	ENSG00000198700
MTRF1	ENSG00000120662
MTRF1L	ENSG00000112031
WIBG	ENSG00000170473
PPAN	ENSG00000130810
FUBP3	ENSG00000107164
FUBP1	ENSG00000162613
KHSRP	ENSG00000088247
CHTOP	ENSG00000160679
PTCD2	ENSG00000049883
POLR2A	ENSG00000181222
MRPL43	ENSG00000055950
DDX55	ENSG00000111364
RPS15	ENSG00000115268
CPSF4	ENSG00000160917
CPSF4L	ENSG00000187959
CSTF2	ENSG00000101811
CSTF2T	ENSG00000177613
RAVER2	ENSG00000162437
RAVER1	ENSG00000161847
EIF1AX	ENSG00000173674
EIF1AY	ENSG00000198692
DALRD3	ENSG00000178149
NSUN2	ENSG00000037474
URB2	ENSG00000135763
PHAX	ENSG00000164902
PPARGC1A	ENSG00000109819
PPARGC1B	ENSG00000155846
PPRC1	ENSG00000148840
PHF5A	ENSG00000100410
MCTS1	ENSG00000232119
RRP7A	ENSG00000189306
DGCR8	ENSG00000128191

ZC3H13	ENSG00000123200
UPF3A	ENSG00000169062
UPF3B	ENSG00000125351
RBM24	ENSG00000112183
RBM38	ENSG00000132819
THOC3	ENSG00000051596
IGHMBP2	ENSG00000132740
PAPD5	ENSG00000121274
PAPD7	ENSG00000112941
GFM2	ENSG00000164347
PHRF1	ENSG00000070047
VARS	ENSG00000096171
VARS2	ENSG00000137411
MRPL1	ENSG00000169288
INTS7	ENSG00000143493
ATXN2L	ENSG00000168488
ATXN2	ENSG00000204842
PPIL4	ENSG00000131013
PPIL3	ENSG00000240344
PPWD1	ENSG00000113593
CWC27	ENSG00000153015
PPIH	ENSG00000171960
NUTF2	ENSG00000102898
MRPL18	ENSG00000112110
LSM11	ENSG00000155858
AARS2	ENSG00000124608
AARS	ENSG00000090861
MIF4GD	ENSG00000125457
CTIF	ENSG00000134030
C9orf129	ENSG00000204352
LUC7L2	ENSG00000269955
LUC7L3	ENSG00000108848
LUC7L	ENSG00000007392
DUS2	ENSG00000167264
RNF17	ENSG00000132972
TDRD1	ENSG00000095627
MRPS10	ENSG00000048544
FAM103A1	ENSG00000169612
ENOX1	ENSG00000120658
ENOX2	ENSG00000165675
RBPMS	ENSG00000157110
RBPMS2	ENSG00000166831
NIP7	ENSG00000132603
RPL3	ENSG00000100316
RPL3L	ENSG00000140986
RNASE11	ENSG00000173464
RNASE12	ENSG00000258436

RPLP0	ENSG00000089157
RBM34	ENSG00000188739
CMTR2	ENSG00000180917
ENDOU	ENSG00000111405
PUM1	ENSG00000134644
PUM2	ENSG00000055917
TYW5	ENSG00000162971
TYW3	ENSG00000162623
TRMT1	ENSG00000104907
RAE1	ENSG00000101146
SLU7	ENSG00000164609
EIF2B4	ENSG00000115211
EIF5B	ENSG00000158417
GPATCH1	ENSG00000076650
RPL36	ENSG00000130255
EEF1A1	ENSG00000156508
EEF1A2	ENSG00000101210
RRP8	ENSG00000132275
UBA52	ENSG00000221983
R3HDM1	ENSG00000048991
R3HDM2	ENSG00000179912
PA2G4	ENSG00000170515
AAR2	ENSG00000131043
ERI1	ENSG00000104626
ERI2	ENSG00000196678
ERI3	ENSG00000117419
NIFK	ENSG00000155438
METTL14	ENSG00000145388
MRPL32	ENSG00000106591
CPSF6	ENSG00000111605
CPSF7	ENSG00000149532
RPL7A	ENSG00000260501
WDR46	ENSG00000227057
SRP14	ENSG00000140319
TFAM	ENSG00000108064
CMSS1	ENSG00000184220
XPO6	ENSG00000169180
EXOSC1	ENSG00000171311
ZNF106	ENSG00000103994
UTP14A	ENSG00000156697
UTP14C	ENSG00000253797
TRUB1	ENSG00000165832
TRUB2	ENSG00000167112
PRIM1	ENSG00000198056
PTCD3	ENSG00000132300
HNRNPA0	ENSG00000177733
HNRNPA1	ENSG00000135486

HNRNPA1L2	ENSG00000139675
HNRNPA2B1	ENSG00000122566
HNRNPA3	ENSG00000170144
HNRNPAB	ENSG00000197451
HNRNPD	ENSG00000138668
HNRNPDL	ENSG00000152795
RANBP2	ENSG00000153201
RPL7	ENSG00000147604
RPL7L1	ENSG00000146223
RNASE13	ENSG00000206150
RPS6	ENSG00000137154
MRPL3	ENSG00000114686
DDX28	ENSG00000182810
DDX47	ENSG00000213782
RNASEH2C	ENSG00000172922
TFB2M	ENSG00000162851
TEP1	ENSG00000129566
NOC4L	ENSG00000184967
CPSF1	ENSG00000071894
DDX1	ENSG00000079785
DDX3X	ENSG00000215301
DDX3Y	ENSG00000067048
DDX4	ENSG00000152670
NOP56	ENSG00000101361
RPS20	ENSG00000008988
MPHOSPH10	ENSG00000124383
MRM1	ENSG00000129282
DXO	ENSG00000236765
DENR	ENSG00000139726
RPL21	ENSG00000122026
RPS10	ENSG00000124614
NAT10	ENSG00000135372
UTP18	ENSG00000011260
NOL12	ENSG00000100101
DDX31	ENSG00000125485
ABCF1	ENSG00000225989
DNMT1	ENSG00000130816
SNRPA1	ENSG00000131876
API5	ENSG00000166181
SHQ1	ENSG00000144736
AKAP1	ENSG00000121057
FTO	ENSG00000140718
PUF60	ENSG00000179950
SAMHD1	ENSG00000101347
RBM45	ENSG00000155636
FAM98B	ENSG00000171262
FAM98A	ENSG00000119812

FAM98C	ENSG00000130244
PCF11	ENSG00000165494
EIF5	ENSG00000100664
ZC3HC1	ENSG00000091732
HNRNPC	ENSG00000092199
HNRNPCL1	ENSG00000179172
RALY	ENSG00000125970
RALYL	ENSG00000184672
SEPSECS	ENSG00000109618
NPM1	ENSG00000181163
NPM3	ENSG00000107833
NPM2	ENSG00000158806
NUPL2	ENSG00000136243
MRPS14	ENSG00000120333
SF3A1	ENSG00000099995
EIF3A	ENSG00000107581
FASTKD5	ENSG00000215251
TDRD3	ENSG00000083544
SNRPB	ENSG00000125835
SNRPN	ENSG00000128739
YRDC	ENSG00000196449
POLR2K	ENSG00000147669
ERN2	ENSG00000134398
ERN1	ENSG00000178607
RPL5	ENSG00000122406
RPS17	ENSG00000184779
RPS17L	ENSG00000182774
DARS	ENSG00000115866
IPO7	ENSG00000205339
IPO8	ENSG00000133704
SMN1	ENSG00000172062
SMN2	ENSG00000205571
GTPBP4	ENSG00000107937
MRPL13	ENSG00000172172
MRPL46	ENSG00000259494
LSM5	ENSG00000106355
EIF4G3	ENSG00000075151
EIF4G1	ENSG00000114867
EIF4G2	ENSG00000110321
GTF2F1	ENSG00000125651
RPS15A	ENSG00000134419
PIH1D2	ENSG00000262740
TTF2	ENSG00000116830
PUS3	ENSG00000110060
RBM7	ENSG00000076053
RBM11	ENSG00000185272
TSEN2	ENSG00000154743

RBM42	ENSG00000126254
UTP3	ENSG00000132467
HNRNPL	ENSG00000104824
HNRNPLL	ENSG00000143889
PTBP1	ENSG00000011304
PTBP2	ENSG00000117569
PTBP3	ENSG00000119314
SYMPK	ENSG00000125755
POLR2F	ENSG00000100142
TSR2	ENSG00000158526
KIAA0391	ENSG00000100890
POLR2G	ENSG00000168002
WDR5	ENSG00000196363
SNRNP40	ENSG00000060688
WDR83	ENSG00000123154
ZFC3H1	ENSG00000133858
RPL15	ENSG00000174748
SNRNP35	ENSG00000184209
SNRNP70	ENSG00000104852
MRPL39	ENSG00000154719
TLR7	ENSG00000196664
TLR8	ENSG00000101916
TLR3	ENSG00000164342
NAA38	ENSG00000128534
PRPF31	ENSG00000105618
MRPL55	ENSG00000162910
TRMT2A	ENSG00000099899
TRMT2B	ENSG00000188917
RPS3A	ENSG00000145425
BRCA1	ENSG00000012048
RIOK2	ENSG00000058729
DHX16	ENSG00000206486
DHX33	ENSG00000005100
DHX35	ENSG00000101452
DHX32	ENSG00000089876
DQX1	ENSG00000144045
DHX15	ENSG00000109606
DHX8	ENSG00000067596
MRPS5	ENSG00000144029
SAP18	ENSG00000150459
TUFM	ENSG00000178952
EEFSEC	ENSG00000132394
TDRD12	ENSG00000173809
SKIV2L2	ENSG00000039123
TRDMT1	ENSG00000107614
ZNF473	ENSG00000142528
FCF1	ENSG00000119616

URB1	ENSG00000142207
CARS	ENSG00000110619
CARS2	ENSG00000134905
CELF1	ENSG00000149187
CELF2	ENSG00000048740
CELF3	ENSG00000159409
CELF4	ENSG00000101489
CELF5	ENSG00000161082
CELF6	ENSG00000140488
XPO1	ENSG00000082898
DDX17	ENSG00000100201
DDX46	ENSG00000145833
DDX5	ENSG00000263077
DDX42	ENSG00000198231
DDX43	ENSG00000080007
DDX53	ENSG00000184735
FIP1L1	ENSG00000145216
MRPL14	ENSG00000180992
TXNL4A	ENSG00000141759
TXNL4B	ENSG00000140830
RBM39	ENSG00000131051
RBM23	ENSG00000100461
CPEB1	ENSG00000214575
CPEB2	ENSG00000137449
CPEB3	ENSG00000107864
CPEB4	ENSG00000113742
INTS9	ENSG00000104299
CACTIN	ENSG00000105298
MPHOSPH6	ENSG00000135698
RPL34	ENSG00000109475
OBFC1	ENSG00000107960
RPS12	ENSG00000112306
PRPF8	ENSG00000174231
SECISBP2L	ENSG00000138593
SECISBP2	ENSG00000187742
RPUSD3	ENSG00000156990
RPUSD4	ENSG00000165526
DDX6	ENSG00000269612
RPL28	ENSG00000108107
ELAC1	ENSG00000141642
NOM1	ENSG00000146909
TROVE2	ENSG00000116747
EEF1E1	ENSG00000124802
TARSL2	ENSG00000185418
TARS	ENSG00000113407
TARS2	ENSG00000143374
IARS	ENSG00000196305

RPL18A	ENSG00000105640
ESF1	ENSG00000089048
EEF2	ENSG00000167658
EFTUD2	ENSG00000108883
DDX60	ENSG00000137628
DDX60L	ENSG00000181381
MRPL2	ENSG00000112651
SLC4A1AP	ENSG00000163798
MAZ	ENSG00000103495
EIF2S3L	ENSG00000180574
EIF2S3	ENSG00000130741
AIMP1	ENSG00000164022
APEX1	ENSG00000100823
EIF3J	ENSG00000104131
PABPC3	ENSG00000151846
PABPC1L	ENSG00000101104
PABPC1L2A	ENSG00000268574
PABPC1L2B	ENSG00000267891
PABPC4L	ENSG00000254535
PABPC5	ENSG00000174740
PABPC4	ENSG00000090621
PABPC1	ENSG00000070756
MEX3A	ENSG00000254726
MEX3B	ENSG00000183496
MEX3C	ENSG00000176624
MEX3D	ENSG00000181588
GEMIN7	ENSG00000142252
MRPS23	ENSG00000181610
PSIP1	ENSG00000164985
SRP54	ENSG00000100883
FARSB	ENSG00000116120
SPEN	ENSG00000065526
RBM15B	ENSG00000179837
RBM15	ENSG00000162775
DNAAF2	ENSG00000165506
SF3B1	ENSG00000115524
RNASEH2B	ENSG00000136104
FARSA	ENSG00000179115
NOB1	ENSG00000141101
EIF2A	ENSG00000144895
RNASEL	ENSG00000135828
TRNT1	ENSG00000072756
EIF4ENIF1	ENSG00000184708
MECP2	ENSG00000169057
RBM22	ENSG00000086589
CMTR1	ENSG00000137200
RPL39	ENSG00000198918

RPL39L	ENSG00000163923
RPSA	ENSG00000168028
PARP4	ENSG00000102699
CSTF1	ENSG00000101138
NCBP1	ENSG00000136937
SNRNP25	ENSG00000161981
DNAJC21	ENSG00000168724
CWC22	ENSG00000163510
NFX1	ENSG00000086102
MRPS31	ENSG00000102738
SMG5	ENSG00000198952
SART1	ENSG00000175467
GEMIN2	ENSG00000092208
NR0B1	ENSG00000169297
DCP2	ENSG00000172795
TAF15	ENSG00000172660
EWSR1	ENSG00000182944
FUS	ENSG00000089280
EDC4	ENSG00000038358
FBLL1	ENSG00000188573
FBL	ENSG00000105202
MRPS27	ENSG00000113048
ALKBH1	ENSG00000100601
ZCCHC17	ENSG00000121766
MRPL49	ENSG00000149792
RPL18	ENSG00000063177
RPS4X	ENSG00000198034
RPS4Y1	ENSG00000129824
RPS4Y2	ENSG00000157828
NOP14	ENSG00000087269
KRR1	ENSG00000111615
NCL	ENSG00000115053
PRPF18	ENSG00000165630
C1D	ENSG00000197223
ADAT3	ENSG00000213638
UTP20	ENSG00000120800
SNIP1	ENSG00000163877
GUF1	ENSG00000151806
GCFC2	ENSG00000005436
UTP6	ENSG00000108651
SRSF1	ENSG00000136450
SRSF3	ENSG00000112081
SRSF4	ENSG00000116350
SRSF5	ENSG00000100650
SRSF6	ENSG00000124193
SRSF7	ENSG00000115875
SRSF9	ENSG00000111786

USB1	ENSG00000103005
NOL6	ENSG00000165271
PPIE	ENSG00000084072
RRBP1	ENSG00000125844
SUZ12	ENSG00000178691
TERT	ENSG00000164362
MSL3	ENSG00000005302
BICC1	ENSG00000122870
MRPS16	ENSG00000182180
RBMX2	ENSG00000134597
MRPL40	ENSG00000185608
ISG20	ENSG00000172183
ISG20L2	ENSG00000143319
AEN	ENSG00000181026
REXO4	ENSG00000148300
TGS1	ENSG00000137574
ZC3H4	ENSG00000130749
ZC3H6	ENSG00000188177
ZC3H8	ENSG00000144161
RPL27A	ENSG00000166441
RPS19	ENSG00000105372
SYF2	ENSG00000117614
TFB1M	ENSG00000029639
DIMT1	ENSG00000086189
TSEN34	ENSG00000170892
DCAF13	ENSG00000164934
RPL9	ENSG00000163682
ARHGEF28	ENSG00000214944
AC004381.6	ENSG00000005189
EIF4H	ENSG00000263344
EIF4B	ENSG00000063046
DDX54	ENSG00000123064
MRPS36	ENSG00000134056
PRPF40A	ENSG00000196504
PRPF40B	ENSG00000110844
EIF3L	ENSG00000100129
NOL8	ENSG00000198000
MTO1	ENSG00000135297
PSMA1	ENSG00000129084
HTATSF1	ENSG00000102241
FTSJ1	ENSG00000269875
LAS1L	ENSG00000001497
POP1	ENSG00000104356
DZIP3	ENSG00000198919
DGCR14	ENSG00000100056
CDK9	ENSG00000136807
PAN3	ENSG00000152520

RPL29	ENSG00000162244
DCPS	ENSG00000110063
MRP63	ENSG00000173141
MRPL27	ENSG00000108826
POLR2H	ENSG00000163882
ZNF239	ENSG00000196793
XRN1	ENSG00000114127
ZFR	ENSG00000056097
ZFR2	ENSG00000105278
AGFG1	ENSG00000173744
NSA2	ENSG00000164346
DDX10	ENSG00000178105
TBL3	ENSG00000183751
INTS2	ENSG00000108506
YBX1	ENSG00000065978
YBX2	ENSG00000006047
YBX3	ENSG00000060138
SURF6	ENSG00000261306
THOC6	ENSG00000131652
EIF3I	ENSG00000084623
STRAP	ENSG00000023734
RPL13	ENSG00000167526
RRP12	ENSG00000052749
MRPS2	ENSG00000122140
ZCCHC8	ENSG00000033030
EFTUD1	ENSG00000140598
RPL35	ENSG00000136942
NSRP1	ENSG00000126653
RPUSD2	ENSG00000166133
BCDIN3D	ENSG00000186666
THUMPD1	ENSG00000066654
TRIM21	ENSG00000132109
RANBP17	ENSG00000204764
XPO7	ENSG00000130227
TRMT12	ENSG00000183665
ACIN1	ENSG00000100813
ZNF385A	ENSG00000161642
RPL23	ENSG00000125691
EED	ENSG00000074266
DNTTIP2	ENSG00000067334
SBDS	ENSG00000126524
DDX51	ENSG00000185163
RBM18	ENSG00000119446
IPO11	ENSG00000086200
RPL22	ENSG00000116251
RPL22L1	ENSG00000163584
LCMT2	ENSG00000168806

ZC3H11A	ENSG00000058673
FYTTD1	ENSG00000122068
RNPS1	ENSG00000205937
RPS23	ENSG00000186468
RNASEH2A	ENSG00000104889
FRG1B	ENSG00000149531
FRG1	ENSG00000260380
NOL7	ENSG00000225921
PRPF38A	ENSG00000134748
RBM41	ENSG00000089682
RBM12	ENSG00000244462
RBM12B	ENSG00000183808
ESRP1	ENSG00000104413
ESRP2	ENSG00000103067
HNRNPF	ENSG00000169813
HNRNPH1	ENSG00000169045
HNRNPH2	ENSG00000126945
HNRNPH3	ENSG00000096746
GRSF1	ENSG00000132463
RNH1	ENSG00000023191
NOL9	ENSG00000162408
SRFBP1	ENSG00000151304
ARL6IP4	ENSG00000182196
RPP40	ENSG00000124787
ALKBH5	ENSG00000091542
SMG7	ENSG00000116698
PDE12	ENSG00000174840
CCRN4L	ENSG00000151014
ANGEL1	ENSG00000013523
ANGEL2	ENSG00000174606
CNOT6	ENSG00000113300
CNOT6L	ENSG00000138767
PDCD11	ENSG00000148843
RPL35A	ENSG00000182899
MRPL21	ENSG00000197345
SMG6	ENSG00000070366
PRPF19	ENSG00000110107
PRPF4	ENSG00000136875
INTS4	ENSG00000149262
SRPK2	ENSG00000135250
SUPV3L1	ENSG00000156502
RBM17	ENSG00000134453
NAF1	ENSG00000145414
YARS	ENSG00000134684
LRRFIP1	ENSG00000124831
LRRFIP2	ENSG00000093167
EIF4E2	ENSG00000135930

EIF4E1B	ENSG00000175766
EIF4E3	ENSG00000163412
EIF4E	ENSG00000151247
BUD13	ENSG00000137656
CTU1	ENSG00000142544
GTPBP1	ENSG00000100226
GTPBP2	ENSG00000172432
MTERFD2	ENSG00000122085
SRPR	ENSG00000182934
DDX50	ENSG00000107625
DDX21	ENSG00000165732
ZCRB1	ENSG00000139168
RPL13A	ENSG00000142541
SRA1	ENSG00000213523
PAPOLB	ENSG00000218823
PAPOLG	ENSG00000115421
PAPOLA	ENSG00000090060
RPL41	ENSG00000229117
ZNF768	ENSG00000169957
SCAF1	ENSG00000126461
MRPS15	ENSG00000116898
RNASET2	ENSG00000026297
EIF2B1	ENSG00000111361
RPL36A	ENSG00000269315
RPL36AL	ENSG00000165502
DDX26B	ENSG00000165359
INTS6	ENSG00000102786
RPS7	ENSG00000171863
RRNAD1	ENSG00000143303
MRPS21	ENSG00000187145
SETD7	ENSG00000145391
RBM8A	ENSG00000131795
MAGOH	ENSG00000162385
MAGOHB	ENSG00000111196
RPL37A	ENSG00000197756
NOLC1	ENSG00000166197
IARS2	ENSG00000067704
LSM1	ENSG00000175324
SUPT4H1	ENSG00000213246
EXO1	ENSG00000174371
DCP1B	ENSG00000151065
DCP1A	ENSG00000162290
DNAJC17	ENSG00000104129
RNGTT	ENSG00000111880
DUSP11	ENSG00000144048
CHERP	ENSG00000085872
WDR61	ENSG00000140395

NANOS1	ENSG00000188613
NANOS2	ENSG00000188425
NANOS3	ENSG00000187556
EBNA1BP2	ENSG00000117395
HELZ	ENSG00000198265
RNMTL1	ENSG00000171861
PDCD4	ENSG00000150593
PTRH2	ENSG00000141378
SART3	ENSG00000075856
ZC3H10	ENSG00000135482
LSM2	ENSG00000224979
MRPS25	ENSG00000131368
RPP21	ENSG00000241863
ZMAT2	ENSG00000146007
IFIT1	ENSG00000185745
IFIT2	ENSG00000119922
IFIT5	ENSG00000152778
IFIT1B	ENSG00000204010
IFIT3	ENSG00000119917
NOVA1	ENSG00000139910
NOVA2	ENSG00000104967
SNRPD3	ENSG00000100028
YARS2	ENSG00000139131
PTCD1	ENSG00000106246
TSR1	ENSG00000167721
MRPS6	ENSG00000243927
TAF9	ENSG00000085231
RPL11	ENSG00000142676
DDX23	ENSG00000174243
DDX59	ENSG00000118197
CCNT2	ENSG00000082258
CCNT1	ENSG00000129315
RPL8	ENSG00000161016
SARNP	ENSG00000205323
IMP3	ENSG00000177971
SRRM4	ENSG00000139767
CNP	ENSG00000173786
LIN28A	ENSG00000131914
LIN28B	ENSG00000187772
EIF3K	ENSG00000178982
SF3B14	ENSG00000115128
EIF2S2	ENSG00000125977
RNMT	ENSG00000101654
MRPL42	ENSG00000198015
THRAP3	ENSG00000054118
BCLAF1	ENSG00000029363
CXorf23	ENSG00000173681

PNO1	ENSG00000115946
RBM4	ENSG00000173933
RBM4B	ENSG00000173914
RBM14	ENSG00000239306
R3HCC1	ENSG00000104679
R3HCC1L	ENSG00000166024
KIAA0430	ENSG00000166783
POP5	ENSG00000167272
MRPL51	ENSG00000111639
MARS	ENSG00000166986
TYW1	ENSG00000198874
MRPL16	ENSG00000166902
DUS1L	ENSG00000169718
QRSL1	ENSG00000130348
WARS2	ENSG00000116874
WRAP53	ENSG00000141499
RSL24D1	ENSG00000137876
SF3B4	ENSG00000143368
TDRD7	ENSG00000196116
LARP1	ENSG00000155506
LARP1B	ENSG00000138709
RPL23A	ENSG00000198242
BAZ2A	ENSG00000076108
BAZ2B	ENSG00000123636
EIF3E	ENSG00000104408
CWC15	ENSG00000261974
TOP1	ENSG00000198900
RPL31	ENSG00000071082
SUPT6H	ENSG00000109111
TST	ENSG00000128311
HNRNPU	ENSG00000153187
HNRNPUL1	ENSG00000105323
HNRNPUL2	ENSG00000214753
RPS25	ENSG00000118181
MRPL23	ENSG00000214026
EIF1AD	ENSG00000175376
SETX	ENSG00000107290
MARS2	ENSG00000247626
CNOT3	ENSG00000088038
MTIF2	ENSG00000085760
JAKMIP1	ENSG00000152969
FASTKD1	ENSG00000138399

Supplemental Table S3	
RBDB database	
Gene Name	Gene ID
RBM5	ENSG00000003756
RBM6	ENSG00000004534
YBX2	ENSG00000006047
CSDE1	ENSG00000009307
PTBP1	ENSG00000011304
ZC3H3	ENSG00000014164
MATR3	ENSG00000015479
SAMD4A	ENSG00000020577
YTHDC2	ENSG00000047188
CUGBP2	ENSG00000048740
PUM2	ENSG00000055917
RC3H2	ENSG00000056586
ZC3H11A	ENSG00000058673
PARP12	ENSG00000059378
CSDA	ENSG00000060138
SFRS8	ENSG00000061936
EIF4B	ENSG00000063046
U2AF2	ENSG00000063244
SFRS14	ENSG00000064607
SPEN	ENSG00000065526
ZC3H15	ENSG00000065548
YBX1	ENSG00000065978
ELAVL1	ENSG00000066044
THUMPD1	ENSG00000066654
DHX8	ENSG00000067596
SRBD1	ENSG00000068784
PABPC1	ENSG00000070756
DAZAP1	ENSG00000071626
IGF2BP2	ENSG00000073792
ZNF638	ENSG00000075292
SART3	ENSG00000075856
MKRN2	ENSG00000075975
RBM7	ENSG00000076053
RBMS2	ENSG00000076067
MBNL3	ENSG00000076770
SNRPA	ENSG00000077312
SYNJ2	ENSG00000078269
A2BP1	ENSG00000078328
	ENSG00000078687
DDX43	ENSG00000080007
KIAA0020	ENSG00000080608
CNOT4	ENSG00000080802
YTHDC1	ENSG00000083896
PPIE	ENSG00000084072

CHERP	ENSG00000085872
RBM22	ENSG00000086589
KHSRP	ENSG00000088247
FUS	ENSG00000089280
RBM41	ENSG00000089682
PCBP4	ENSG00000090097
PABPC4	ENSG00000090621
TNRC6A	ENSG00000090905
RBM27	ENSG00000091009
HNRNPC	ENSG00000092199
DAZL	ENSG00000092345
HNRNPH3	ENSG00000096746
SETD1A	ENSG00000099381
CIRBP	ENSG00000099622
HNRNPM	ENSG00000099783
TRMT2A	ENSG00000099899
SF3A1	ENSG00000099995
SNRPD3	ENSG00000100028
POLDIP3	ENSG00000100227
ZMAT5	ENSG00000100319
RBM9	ENSG00000100320
ZC3H7B	ENSG00000100403
RBM23	ENSG00000100461
SFRS5	ENSG00000100650
ZC3H14	ENSG00000100722
ACIN1	ENSG00000100813
PABPN1	ENSG00000100836
PABPC1L	ENSG00000101104
BRUNOL4	ENSG00000101489
CSTF2	ENSG00000101811
ZC3H12B	ENSG00000102053
FMR1	ENSG00000102081
HTATSF1	ENSG00000102241
RBM3	ENSG00000102317
ESRP2	ENSG00000103067
MTHFSD	ENSG00000103248
DNAJC17	ENSG00000104129
MYEF2	ENSG00000104177
ESRP1	ENSG00000104413
HNRNPL	ENSG00000104824
SNRNP70	ENSG00000104852
SFRS16	ENSG00000104859
TRMT1	ENSG00000104907
NOVA2	ENSG00000104967
SF4	ENSG00000105705
ZC3HAV1	ENSG00000105939
EIF3B	ENSG00000106263

RBM28	ENSG00000106344
LSM5	ENSG00000106355
EIF4H	ENSG00000106682
ELAVL2	ENSG00000107105
FUBP3	ENSG00000107164
CPEB3	ENSG00000107864
LARP4B	ENSG00000107929
SUPT6H	ENSG00000109111
PPARGC1A	ENSG00000109819
CPSF6	ENSG00000111605
KRR1	ENSG00000111615
SFRS9	ENSG00000111786
SFRS3	ENSG00000112081
RBM24	ENSG00000112183
KHDRBS2	ENSG00000112232
QKI	ENSG00000112531
CPEB4	ENSG00000113742
FXR1	ENSG00000114416
NCL	ENSG00000115053
	ENSG00000115128
HDLBP	ENSG00000115677
SFRS7	ENSG00000115875
PNO1	ENSG00000115946
TIA1	ENSG00000116001
SFRS4	ENSG00000116350
SFPQ	ENSG00000116560
SFRS11	ENSG00000116754
PRPF3	ENSG00000117360
PTBP2	ENSG00000117569
ROD1	ENSG00000119314
RBM18	ENSG00000119446
C14orf156	ENSG00000119705
RBM25	ENSG00000119707
ENOX1	ENSG00000120658
TARDBP	ENSG00000120948
AKAP1	ENSG00000121057
PSPC1	ENSG00000121390
ZCCHC17	ENSG00000121766
KHDRBS1	ENSG00000121774
ZC3H7A	ENSG00000122299
HNRNPA2B1	ENSG00000122566
BICC1	ENSG00000122870
RBM19	ENSG00000122965
ZC3H13	ENSG00000123200
SFRS6	ENSG00000124193
RNF113A	ENSG00000125352
SNRPD2	ENSG00000125743

SNRPB	ENSG00000125835
SNRPB2	ENSG00000125870
HNRNPR	ENSG00000125944
RALY	ENSG00000125970
RBM42	ENSG00000126254
HNRNPH2	ENSG00000126945
ZFP36	ENSG00000128016
NAA38	ENSG00000128534
SNRPN	ENSG00000128739
FXR2	ENSG00000129245
SAFB2	ENSG00000130254
LSM7	ENSG00000130332
LSM4	ENSG00000130520
ZC3H4	ENSG00000130749
EIF3G	ENSG00000130811
DKC1	ENSG00000130826
PPIL4	ENSG00000131013
RBM39	ENSG00000131051
ANKHD1	ENSG00000131503
KHDRBS3	ENSG00000131773
RBM8A	ENSG00000131795
LIN28	ENSG00000131914
GRSF1	ENSG00000132463
ANKRD17	ENSG00000132466
UNK	ENSG00000132478
NIP7	ENSG00000132603
TOE1	ENSG00000132773
RBM38	ENSG00000132819
SRRM1	ENSG00000133226
MKRN1	ENSG00000133606
EIF2S1	ENSG00000134001
THUMPD3	ENSG00000134077
RBMX2	ENSG00000134597
PUM1	ENSG00000134644
MSI1	ENSG00000135097
SYNCRIP	ENSG00000135316
ZC3H10	ENSG00000135482
HNRNPA1	ENSG00000135486
RC3H1	ENSG00000135870
IGF2BP3	ENSG00000136231
NUPL2	ENSG00000136243
SFRS1	ENSG00000136450
TRA2B	ENSG00000136527
CPEB2	ENSG00000137449
ALKBH8	ENSG00000137760
SLTM	ENSG00000137776
PNPT1	ENSG00000138035

THUMPD2	ENSG00000138050
ASCC1	ENSG00000138303
SSB	ENSG00000138385
HNRNPD	ENSG00000138668
LARP1B	ENSG00000138709
G3BP2	ENSG00000138757
ZCRB1	ENSG00000139168
SNRPF	ENSG00000139343
	ENSG00000139675
SETD1B	ENSG00000139718
RBM26	ENSG00000139746
MBNL2	ENSG00000139793
RNF113B	ENSG00000139797
NOVA1	ENSG00000139910
BRUNOL6	ENSG00000140488
DUS3L	ENSG00000141994
SF3B4	ENSG00000143368
LGTN	ENSG00000143486
HNRPLL	ENSG00000143889
SNRPG	ENSG00000143977
ZC3H8	ENSG00000144161
RBMS3	ENSG00000144642
G3BP1	ENSG00000145907
NONO	ENSG00000147140
RBMX	ENSG00000147274
A1CF	ENSG00000148584
PPRC1	ENSG00000148840
PDCD11	ENSG00000148843
TUT1	ENSG00000149016
CUGBP1	ENSG00000149187
RPS3	ENSG00000149273
ZC3H12C	ENSG00000149289
CPSF7	ENSG00000149532
YTHDF1	ENSG00000149658
PABPC3	ENSG00000151846
TIAL1	ENSG00000151923
RBM46	ENSG00000151962
UHMK1	ENSG00000152332
BOLL	ENSG00000152430
ZFP36L2	ENSG00000152518
PAN3	ENSG00000152520
MBNL1	ENSG00000152601
HNRPDL	ENSG00000152795
CARHSP1	ENSG00000153048
RBMS1	ENSG00000153250
SFRS12	ENSG00000153914
MSI2	ENSG00000153944

SFRS13B	ENSG00000154548
C4orf23	ENSG00000155275
MKI67IP	ENSG00000155438
LARP1	ENSG00000155506
RBM45	ENSG00000155636
PPARGC1B	ENSG00000155846
LSM11	ENSG00000155858
SFRS15	ENSG00000156304
RBPMS	ENSG00000157110
ZC3H18	ENSG00000158545
SYNJ1	ENSG00000159082
IGF2BP1	ENSG00000159217
TNRC4	ENSG00000159409
U2AF1	ENSG00000160201
SAFB	ENSG00000160633
CPSF4	ENSG00000160917
BRUNOL5	ENSG00000161082
U2AF1L4	ENSG00000161265
SFRS2	ENSG00000161547
LARP4	ENSG00000161813
RAVER1	ENSG00000161847
ELAVL4	ENSG00000162374
RAVER2	ENSG00000162437
FUBP1	ENSG00000162613
RBM15	ENSG00000162775
DHX57	ENSG00000163214
TDRD10	ENSG00000163239
TIPARP	ENSG00000163659
RBM47	ENSG00000163694
SR140	ENSG00000163714
TTC14	ENSG00000163728
LSM6	ENSG00000164167
TRA2A	ENSG00000164548
HNRNPK	ENSG00000165119
ENOX2	ENSG00000165675
LARP6	ENSG00000166173
KIAA0430	ENSG00000166783
RBPMS2	ENSG00000166831
SNRPD1	ENSG00000167088
SAMD14	ENSG00000167100
	ENSG00000167281
POLR2G	ENSG00000168002
SF1	ENSG00000168066
HNRNPH1	ENSG00000169045
ZRSR2	ENSG00000169249
PCBP1	ENSG00000169564
RBMY1F	ENSG00000169800

HNRNPF	ENSG00000169813
HNRNPA3	ENSG00000170144
RBMXL2	ENSG00000170748
LSM3	ENSG00000170860
NCBP2L	ENSG00000170935
CSDC2	ENSG00000172346
TAF15	ENSG00000172660
RBM4B	ENSG00000173914
RBM4	ENSG00000173933
LARP7	ENSG00000174720
PABPC5	ENSG00000174740
LSM1	ENSG00000175324
RBMXL3	ENSG00000175718
MEX3C	ENSG00000176624
RBM44	ENSG00000177483
CSTF2T	ENSG00000177613
HNRNPA0	ENSG00000177733
ZC3H12D	ENSG00000178199
SAMD4B	ENSG00000179134
HNRNPCL1	ENSG00000179172
MKRN3	ENSG00000179455
RBM15B	ENSG00000179837
PUF60	ENSG00000179950
TRNAU1AP	ENSG00000180098
SFRS2B	ENSG00000180771
MEX3D	ENSG00000181588
LSM10	ENSG00000181817
SNRPE	ENSG00000182004
TDRKH	ENSG00000182134
RBM10	ENSG00000182872
LENG9	ENSG00000182909
EWSR1	ENSG00000182944
LSMD1	ENSG00000183011
DND1	ENSG00000183403
MEX3B	ENSG00000183496
PCBP3	ENSG00000183570
THOC4	ENSG00000183684
RBM12B	ENSG00000183808
SNRNP35	ENSG00000184209
PABPC1L2B	ENSG00000184388
RALYL	ENSG00000184672
DDX53	ENSG00000184735
RBM33	ENSG00000184863
RBM43	ENSG00000184898
RBM11	ENSG00000185272
ZFP36L1	ENSG00000185650
YTHDF3	ENSG00000185728

RNPC3	ENSG00000185946
PABPC1L2A	ENSG00000186288
DAZ3	ENSG00000187191
RDM1	ENSG00000187456
LIN28B	ENSG00000187772
CPSF4L	ENSG00000187959
DAZ1	ENSG00000188120
ZC3H6	ENSG00000188177
SFRS13A	ENSG00000188529
RBM34	ENSG00000188739
RRP7A	ENSG00000189306
ELAVL3	ENSG00000196361
C14orf21	ENSG00000196943
PCBP2	ENSG00000197111
ZGPAT	ENSG00000197114
HNRNPAB	ENSG00000197451
NOL8	ENSG00000198000
HELZ	ENSG00000198265
YTHDF2	ENSG00000198492
SFMBT2	ENSG00000198879
L3MBTL3	ENSG00000198945
MEX3A	ENSG00000203759
RBM20	ENSG00000203867
DPPA5	ENSG00000203909
MIR1236	ENSG00000204356
	ENSG00000204392
PPP1R10	ENSG00000204569
PRR3	ENSG00000204576
PABPN1L	ENSG00000205022
RNPS1	ENSG00000205937
DAZ2	ENSG00000205944
ZRSR1	ENSG00000212643
RBM16	ENSG00000213079
	ENSG00000213250
RBMXL1	ENSG00000213516
CPEB1	ENSG00000214575
	ENSG00000215042
	ENSG00000215492
RBMY1J	ENSG00000226941
SNRPEL1	ENSG00000227624
	ENSG00000231942
MCTS1	ENSG00000232119
PABPC4L	ENSG00000233034
RBMY1A1	ENSG00000234414
MKRNP5	ENSG00000238222
RBM14	ENSG00000239306
NSUN6	ENSG00000241058

UNKL	ENSG00000242204
RBMY1E	ENSG00000242389
HNRNPA1L2	ENSG00000242466
RBMY1B	ENSG00000242875
RBMY1D	ENSG00000244395
RBM12	ENSG00000244462
DAZ4	ENSG00000205916
DGKQ	ENSG00000145214
RBM17	ENSG00000134453
MCM3AP	ENSG00000160294
PARN	ENSG00000140694
TLR2	ENSG00000137462
PARP10	ENSG00000178685
MOV10L1	ENSG00000073146
CCAR1	ENSG00000060339
LEMD3	ENSG00000174106
UPF3B	ENSG00000125351
RCAN2	ENSG00000172348
SRRT	ENSG00000087087
ZC3H12A	ENSG00000163874
ZC3HAV1L	ENSG00000146858
PARP14	ENSG00000173193
TMEM63A	ENSG00000196187
SLBP	ENSG00000163950
GTF3A	ENSG00000122034
NUFIP1	ENSG00000083635
SRRM2	ENSG00000167978
APTX	ENSG00000137074
ZFR	ENSG00000056097
UPF1	ENSG00000005007
KIN	ENSG00000151657
ZNF239	ENSG00000196793
ZNF74	ENSG00000185252
SNRPC	ENSG00000124562
C1orf25	ENSG00000121486
ZFR2	ENSG00000105278
IREB2	ENSG00000136381
ACO1	ENSG00000122729
TROVE2	ENSG00000116747
TEP1	ENSG00000129566
GAPDH	ENSG00000111640
ZRANB2	ENSG00000132485

Supplemental Table S4
Intersected genes
Gene ID
ENSG00000003756
ENSG00000004534
ENSG00000006047
ENSG00000009307
ENSG00000011304
ENSG00000014164
ENSG00000015479
ENSG00000020577
ENSG00000047188
ENSG00000048740
ENSG00000055917
ENSG00000056586
ENSG00000058673
ENSG00000059378
ENSG00000060138
ENSG00000061936
ENSG00000063046
ENSG00000063244
ENSG00000064607
ENSG00000065526
ENSG00000065548
ENSG00000065978
ENSG00000066044
ENSG00000066654
ENSG00000067596
ENSG00000068784
ENSG00000070756
ENSG00000071626
ENSG00000073792
ENSG00000075292
ENSG00000075856
ENSG00000075975
ENSG00000076053
ENSG00000076067
ENSG00000076770
ENSG00000077312
ENSG00000078328
ENSG00000078687
ENSG00000080007
ENSG00000080608
ENSG00000080802
ENSG00000084072
ENSG00000085872
ENSG00000086589
ENSG00000088247
ENSG00000089280
ENSG00000089682

ENSG00000090097
ENSG00000090621
ENSG00000090905
ENSG00000091009
ENSG00000092199
ENSG00000092345
ENSG00000096746
ENSG00000099381
ENSG00000099622
ENSG00000099783
ENSG00000099899
ENSG00000099995
ENSG00000100028
ENSG00000100227
ENSG00000100319
ENSG00000100320
ENSG00000100403
ENSG00000100461
ENSG00000100650
ENSG00000100722
ENSG00000100813
ENSG00000100836
ENSG00000101104
ENSG00000101489
ENSG00000101811
ENSG00000102053
ENSG00000102081
ENSG00000102241
ENSG00000102317
ENSG00000103067
ENSG00000103248
ENSG00000104129
ENSG00000104177
ENSG00000104413
ENSG00000104824
ENSG00000104852
ENSG00000104859
ENSG00000104907
ENSG00000104967
ENSG00000105705
ENSG00000105939
ENSG00000106263
ENSG00000106344
ENSG00000106355
ENSG00000107105
ENSG00000107164
ENSG00000107864
ENSG00000107929
ENSG00000109111
ENSG00000109819

ENSG00000111605
ENSG00000111615
ENSG00000111786
ENSG00000112081
ENSG00000112183
ENSG00000112232
ENSG00000112531
ENSG00000113742
ENSG00000114416
ENSG00000115053
ENSG00000115128
ENSG00000115677
ENSG00000115875
ENSG00000115946
ENSG00000116001
ENSG00000116350
ENSG00000116560
ENSG00000116754
ENSG00000117569
ENSG00000119314
ENSG00000119446
ENSG00000119705
ENSG00000119707
ENSG00000120658
ENSG00000120948
ENSG00000121057
ENSG00000121390
ENSG00000121766
ENSG00000121774
ENSG00000122299
ENSG00000122566
ENSG00000122870
ENSG00000122965
ENSG00000123200
ENSG00000124193
ENSG00000125352
ENSG00000125743
ENSG00000125835
ENSG00000125870
ENSG00000125944
ENSG00000125970
ENSG00000126254
ENSG00000126945
ENSG00000128016
ENSG00000128534
ENSG00000128739
ENSG00000129245
ENSG00000130254
ENSG00000130332
ENSG00000130520

ENSG00000130749
ENSG00000130811
ENSG00000130826
ENSG00000131013
ENSG00000131051
ENSG00000131503
ENSG00000131773
ENSG00000131795
ENSG00000131914
ENSG00000132463
ENSG00000132466
ENSG00000132478
ENSG00000132603
ENSG00000132773
ENSG00000132819
ENSG00000133226
ENSG00000133606
ENSG00000134001
ENSG00000134077
ENSG00000134597
ENSG00000134644
ENSG00000135097
ENSG00000135316
ENSG00000135482
ENSG00000135486
ENSG00000135870
ENSG00000136231
ENSG00000136243
ENSG00000136450
ENSG00000136527
ENSG00000137449
ENSG00000137760
ENSG00000137776
ENSG00000138035
ENSG00000138050
ENSG00000138303
ENSG00000138385
ENSG00000138668
ENSG00000138709
ENSG00000138757
ENSG00000139168
ENSG00000139343
ENSG00000139675
ENSG00000139718
ENSG00000139746
ENSG00000139793
ENSG00000139797
ENSG00000139910
ENSG00000140488
ENSG00000141994

ENSG00000143368
ENSG00000143486
ENSG00000143889
ENSG00000143977
ENSG00000144161
ENSG00000144642
ENSG00000145907
ENSG00000147140
ENSG00000147274
ENSG00000148584
ENSG00000148840
ENSG00000148843
ENSG00000149016
ENSG00000149187
ENSG00000149273
ENSG00000149289
ENSG00000149532
ENSG00000149658
ENSG00000151846
ENSG00000151923
ENSG00000151962
ENSG00000152332
ENSG00000152430
ENSG00000152518
ENSG00000152520
ENSG00000152601
ENSG00000152795
ENSG00000153048
ENSG00000153250
ENSG00000153914
ENSG00000153944
ENSG00000154548
ENSG00000155275
ENSG00000155438
ENSG00000155506
ENSG00000155636
ENSG00000155846
ENSG00000155858
ENSG00000156304
ENSG00000157110
ENSG00000158545
ENSG00000159217
ENSG00000159409
ENSG00000160201
ENSG00000160633
ENSG00000160917
ENSG00000161082
ENSG00000161265
ENSG00000161547
ENSG00000161813

ENSG00000161847
ENSG00000162374
ENSG00000162437
ENSG00000162613
ENSG00000162775
ENSG00000163214
ENSG00000163239
ENSG00000163659
ENSG00000163694
ENSG00000163714
ENSG00000164167
ENSG00000164548
ENSG00000165119
ENSG00000165675
ENSG00000166173
ENSG00000166783
ENSG00000166831
ENSG00000167088
ENSG00000168002
ENSG00000168066
ENSG00000169045
ENSG00000169249
ENSG00000169564
ENSG00000169800
ENSG00000169813
ENSG00000170144
ENSG00000170748
ENSG00000170860
ENSG00000170935
ENSG00000172346
ENSG00000172660
ENSG00000173914
ENSG00000173933
ENSG00000174720
ENSG00000174740
ENSG00000175324
ENSG00000175718
ENSG00000176624
ENSG00000177483
ENSG00000177613
ENSG00000177733
ENSG00000178199
ENSG00000179134
ENSG00000179172
ENSG00000179455
ENSG00000179837
ENSG00000179950
ENSG00000180098
ENSG00000180771
ENSG00000181588

ENSG00000181817
ENSG00000182004
ENSG00000182134
ENSG00000182872
ENSG00000182944
ENSG00000183011
ENSG00000183496
ENSG00000183570
ENSG00000183684
ENSG00000183808
ENSG00000184209
ENSG00000184672
ENSG00000184735
ENSG00000184863
ENSG00000184898
ENSG00000185272
ENSG00000185650
ENSG00000185728
ENSG00000185946
ENSG00000187191
ENSG00000187456
ENSG00000187772
ENSG00000187959
ENSG00000188120
ENSG00000188177
ENSG00000188739
ENSG00000189306
ENSG00000196361
ENSG00000196943
ENSG00000197111
ENSG00000197114
ENSG00000197451
ENSG00000198000
ENSG00000198265
ENSG00000198492
ENSG00000203867
ENSG00000205022
ENSG00000205937
ENSG00000205944
ENSG00000212643
ENSG00000213079
ENSG00000213516
ENSG00000214575
ENSG00000226941
ENSG00000232119
ENSG00000234414
ENSG00000239306
ENSG00000241058
ENSG00000242389
ENSG00000242875

ENSG00000244395
ENSG00000244462
ENSG00000205916
ENSG00000134453
ENSG00000140694
ENSG00000073146
ENSG00000060339
ENSG00000125351
ENSG00000087087
ENSG00000163874
ENSG00000146858
ENSG00000163950
ENSG00000122034
ENSG00000083635
ENSG00000167978
ENSG00000137074
ENSG00000056097
ENSG00000005007
ENSG00000151657
ENSG00000196793
ENSG00000124562
ENSG00000121486
ENSG00000105278
ENSG00000136381
ENSG00000122729
ENSG00000116747
ENSG00000129566
ENSG00000111640
ENSG00000132485

	Supplemental Table S5								
	Patients data								
	Breast			Colon			Lung		
	Fold	HR-RFS	p-value	Fold	HR-RFS	p-value	Fold	HR-PFS	p-value
**EIF4E3**	-2.5564525	0.53234	0	-4.61976	0.79842	0.13583	-1.81396	0.48695	0.00003
**CPEB1**	-2.1523921	0.59939	0	-3.15939	2.04875	0	-2.38571	1.62977	0.00036
**ZCCHC24**	-3.5232604	0.62021	0	-2.0707	1.43226	0.00344	-1.83131	0.63161	0.00001
**RBMS3**	-5.6088142	0.62889	0	-4.8798	1.76292	0.00068	-2.17077	0.39223	0
**VARS2**	1.5735226	0.67179	0	1.529402	0.53876	0.00111	1.524821	1.49854	0.00309
**RBM20**	-2.4706635	0.72344	0.00017	-4.78688	1.14485	0.39185	-2.12197	0.83619	0.2451
**ZNF239**	2.0658677	0.73694	0	3.124806	0.70262	0.00307	1.782467	1.79384	0
**MSI2**	2.7480783	0.78904	0.00445	2.574227	0.55226	0.00073	2.460587	1.63675	0.0015
**SMAD9**	-2.2608936	0.79047	0.01363	-2.20768	0.71869	0.06027	-2.81383	0.4828	0
**METTL1**	1.7511885	0.80348	0.00013	2.294277	0.68486	0.001	1.690236	1.87869	0
**PTRF**	-4.0403996	0.80427	0.00008	-2.43163	1.74189	0	-1.76285	0.86587	0.19517
**KCTD12**	-3.8652518	0.8185	0.00056	-3.22674	1.28145	0.03044	-1.73692	0.52706	0
**PPARGC1B**	-1.9268708	0.82011	0.01108	-3.05582	0.62334	0.00072	-2.53717	0.59805	0.0007
**CSRP1**	-2.6758204	0.84281	0.00494	-2.62139	2.19912	0	-1.57295	0.59404	0
**TDRKH**	2.3332734	0.87318	0.08573	1.82257	0.80404	0.1335	2.859908	0.75999	0.04739
**DDX43**	-3.4715905	0.88211	0.03074	-2.2704	0.90094	0.40216	-2.16479	0.87977	0.20069
**NCBP2**	2.0674076	0.8914	0.04455	1.78995	0.70218	0.0108	1.600946	0.73028	0.0028
**PPARGC1A**	-5.3454933	0.91793	0.1245	-5.25166	0.73048	0.02729	-3.97437	0.66926	0.00058
**LSM12**	1.7422183	1.096	0.13299	1.660524	0.6384	0.0001	1.521417	0.72419	0.00331
**RANBP17**	1.7121521	1.12989	0.04236	1.738862	0.62177	0.00004	1.780546	1.36201	0.00173
**RBPMS2**	-2.594975	1.17867	0.03901	-4.22149	1.8345	0.00001	-2.85701	0.68175	0.00607
**ZC3H12C**	-2.633514	1.18293	0.04889	-3.34095	0.56872	0.00012	-1.81204	0.52084	0.00008
**CDKN2A**	2.317773	1.21884	0.00084	3.791205	1.54379	0.00051	2.792538	1.32031	0.0075
**CCDC137**	1.8659058	1.32842	0.00028	1.684724	0.76802	0.07674	1.526315	1.54548	0.00568
**BRCA1**	1.967411	1.42034	0	1.84318	0.52604	0	1.652428	1.59155	0
**MEX3A**	2.8456798	1.43011	0	4.859792	1.44525	0.0094	1.980403	1.62685	0.00045
**DKC1**	1.5073702	1.43726	0	2.791593	0.63762	0.00017	1.579109	0.73824	0.00243
**HDGF**	2.8803022	1.44101	0	1.83921	0.77977	0.03315	1.631098	1.69849	0.00001
**VARS**	1.6565965	1.4678	0	1.585115	1.22664	0.08847	1.541602	1.26068	0.02947
**BYSL**	1.7807758	1.49473	0	2.248924	0.82533	0.09083	1.699456	1.69909	0.00001
**NME1**	1.642789	1.54152	0	1.815166	0.58585	0	2.143543	1.91581	0
**EZH2**	4.5005193	1.65722	0	2.346378	0.50185	0	2.223194	1.38317	0.00104
**TUBB2B**	-4.0020413	0.83	0.0008	1.66029	1.31	0.019	1.921708	1.22	0.045
**RNASE1**	-1.7405324	1.42	7.4E-11	-2.03564	1.52	0.00024	1.522226	0.7	0.00034
**PTBP2**	-1.6146665	0.84	0.0071	-1.77519	1.11	0.38	1.50383	0.61	0.000032
**CNOT1**	1.5107083	0.84	0.0028	1.505186	0.85	0.18	1.666359	2	6.9E-12
**TIA1**	1.5232474	0.79	0.00001	1.789311	0.66	0.00062	1.597316	0.53	2.4E-07
**SFN**	1.5314347	1.51	6.5E-13	-2.15481	0.88	0.27	3.499593	1.38	0.002
**UBE2I**	1.552005	0.71	2.7E-09	1.694966	1.23	0.13	1.541654	0.42	7.6E-13
**RBM47**	1.5552849	0.7	1.7E-09	-2.4046	0.65	0.00027	1.51667	0.67	0.00022
**HEATR1**	1.5631014	0.79	0.002	1.965947	0.52	1.3E-07	1.505009	1.4	0.021
**FLNB**	1.582657	0.64	1.7E-15	1.602078	1.31	0.024	1.98022	0.67	0.00044
**SECISBP2L**	1.6482694	1.15	0.023	-3.23646	1.51	0.00036	-1.86501	0.67	0.0012
**RPL39L**	1.6922513	1.54	1.9E-14	1.72875	1.23	0.13	2.247436	1.7	7.3E-08
**GSPT1**	1.7117828	0.65	2.7E-08	1.804728	0.7	0.0026	1.636707	0.49	0.000066
**EPRS**	1.7460929	1.58	1.00E-16	1.511292	0.74	0.015	1.594859	0.63	0.00016
**SSR1**	1.759067	0.78	0.002	1.523605	0.73	0.014	1.740543	0.54	0.00004
**KIAA1324**	1.7619578	0.63	4.4E-16	-3.28008	0.53	5.6E-07	2.993405	0.76	0.01
**SYNCRIP**	1.7750144	1.57	1.00E-16	1.70378	0.7	0.002	1.60214	0.62	0.000056
**SNRPE**	1.8730097	0.82	0.012	1.96655	0.69	0.0044	1.588003	0.55	0.00081
**WDR5**	1.8811277	1.06	0.43	1.970459	0.53	1.3E-07	1.501066	1.3	0.059
**LRRC59**	2.1593196	1.54	1.9E-14	1.662044	0.53	0.000012	1.663421	1.4	0.0033
**APOBEC3B**	2.973276	1.63	1.00E-16	-3.11912	0.87	0.26	1.596806	1.12	0.27
**HIST1H1E**	3.0772972	1.19	0.0011	-1.79113	1.41	0.0037	1.565853	0.78	0.01
**HIST1H1C**	3.1830142	1.33	5.5E-07	-4.37127	1.46	0.0014	1.768493	1.29	0.02
**HIST1H1D**	3.3739192	1.11	0.094	1.664465	0.81	0.084	2.204443	1.52	0.000032
**OASL**	3.648853	0.85	0.0035	-2.21856	1.27	0.068	-1.74194	1.91	3.5E-11
**DQX1**	4.0624804	1.15	0.078	-3.05542	0.78	0.053	1.580043	1.49	0.0051

Supplemental Table S1: The 58 common RBPs signature patient data across breast, colon and lung cancers were identifi

Supplemental Table S6		
Breast cancer (R-Values)		
	R	-LOG10(p-value)
EIF4E3/MEX3A	-0.23691	4
GSPT1/MEX3A	-0.21764	4
SNRPE/GSPT1	-0.20594	4
SMAD9/GSPT1	-0.19957	4
EIF4E3/SNRPE	-0.19416	4
EIF4E3/CDKN2A	-0.19331	4
GSPT1/CDKN2A	-0.1879	3.69897
EIF4E3 / A_23_P406425/vars	-0.16287	2.886057
EIF4E3/PPARGC1B	-0.15633	2.69897
PPARGC1B/GSPT1	-0.1089	1.497573
SMAD9/EIF4E3	-0.09683	1.248721
EIF4E3/TIA1	-0.085	1.02641
SMAD9/SSR1	-0.08395	1.007446
EIF4E3/SSR1	-0.05264	0.5223
SMAD9/SNRPE	-0.03341	0.291494
SMAD9 / A_23_P2814/vars	-0.02983	0.253755
SSR1/GSPT1	0.01646	0.127145
SSR1/MEX3A	0.02253	0.181906
TIA1/GSPT1	0.02577	0.212965
EIF4E3/RPL39L	0.03771	0.33885
SSR1/TIA1	0.03804	0.342657
SSR1/CDKN2A	0.03839	0.346498
EIF4E3/GSPT1	0.05554	0.561458
SMAD9/RPL39L	0.06609	0.713544
SNRPE/CDKN2A	0.07521	0.857924
VARS/CDKN2A	0.07581	0.868061
TIA1/SNRPE	0.07801	0.904831
PPARGC1B/SSR1	0.08625	1.048662
PPARGC1B/SNRPE	0.09211	1.157391
VARS/RPL39L	0.11973	1.739929
VARS/SNRPE	0.1406	2.259637
GSPT1/RPL39L	0.14381	2.346787
SSR1/RPL39L	0.16799	3.045757
SNRPE/RPL39L	0.16839	3.045757
TIA1 / A_24_P417474/vars	0.1715	3.154902
SMAD9/TIA1	0.18397	3.522879
PPARGC1B/RPL39L	0.20654	4
SSR1 / A_32_P128588/vars	0.20987	4
VARS/GSPT1	0.2119	4
PPARGC1B / A_23_P213959/vars	0.21545	4
PPARGC1B/MEX3A	0.22649	4
SMAD9/CDKN2A	0.23195	4
SMAD9/MEX3A	0.23819	4
VARS/MEX3A	0.24795	4
RPL39L/CDKN2A	0.26696	4
MEX3A/RPL39L	0.30362	4
PPARGC1B/CDKN2A	0.30475	4
TIA1/RPL39L	0.30683	4
SMAD9/PPARGC1B	0.31134	4
PPARGC1B/TIA1	0.32469	4
SNRPE/MEX3A	0.35969	4
MEX3A/CDKN2A	0.38055	4
SSR1/SNRPE	0.39687	4
TIA1/CDKN2A	0.40082	4
TIA1/MEX3A	0.5112	4

Supplemental Table S7		
Colon cancer (R-Values)		
	R	log(p-value)
EIF4E3/MEX3A	-0.47469	4
SSR1/RPL39L	-0.45021	4
PPARGC1B/CDKN2A	-0.44132	4
TIA1/CDKN2A	-0.29916	2.638272
EIF4E3/PPARGC1B	-0.26553	2.154902
CDKN2A/MEX3A	-0.23762	1.790485
SMAD9/CDKN2A	-0.22338	1.619789
GSPT1/RPL39L	-0.21954	1.575118
EIF4E3/SNRPE	-0.21226	1.492144
GSPT1/TIA1	-0.20988	1.465974
VARS/TIA1	-0.1722	1.078314
GSPT1/SNRPE	-0.16335	0.996109
EIF4E3/SMAD9	-0.13858	0.783043
EIF4E3/VARS	-0.13627	0.764472
SMAD9/SSR1	-0.11126	0.575772
SSR1/SNRPE	-0.0874	0.417482
SSR1/MEX3A	-0.0677	0.301899
RPL39L/CDKN2A	-0.04491	0.184422
SMAD9/VARS	-0.04483	0.184024
SMAD9/RPL39L	-0.02111	0.079251
EIF4E3/GSPT1	-0.01822	0.067628
SSR1/CDKN2A	-0.01554	0.057099
SNRPE/CDKN2A	-0.01343	0.048954
PPARGC1B/SNRPE	-0.00188	0.006564
GSPT1/CDKN2A	-0.00046	0.00161
SMAD9/GSPT1	0.01666	0.06148
VARS/CDKN2A	0.01675	0.061831
VARS/SNRPE	0.02575	0.098378
SMAD9/TIA1	0.0271	0.104136
SSR1/TIA1	0.02836	0.109467
PPARGC1B/SSR1	0.03722	0.148558
RPL39L/TIA1	0.04203	0.170761
SNRPE/TIA1	0.0631	0.276791
RPL39L/MEX3A	0.06394	0.281415
SMAD9/SNRPE	0.077	0.354774
PPARGC1B/RPL39L	0.09385	0.458296
VARS/MEX3A	0.09994	0.49812
SNRPE/MEX3A	0.10221	0.513286
EIF4E3/CDKN2A	0.10453	0.529002
SMAD9/MEX3A	0.10784	0.551912
VARS/RPL39L	0.11038	0.569602
GSPT1/MEX3A	0.13142	0.725842
PPARGC1B/VARS	0.18889	1.241845
PPARGC1B/TIA1	0.20192	1.378824
PPARGC1B/SMAD9	0.20255	1.385103
VARS/GSPT1	0.23713	1.785156
VARS/SSR1	0.2685	2.19382
PPARGC1B/GSPT1	0.28158	2.387216
EIF4E3/SSR1	0.2835	2.408935
PPARGC1B/MEX3A	0.30232	2.69897
RPL39L/SNRPE	0.32058	3
TIA1/MEX3A	0.32473	3.045757
GSPT1/SSR1	0.33106	3.154902

Supplemental Table S8		
Lung cancer (R-Values)		
	R	log(p-value)
SMAD9/SSR1	-0.30175	4
EIF4E3/MEX3A	-0.25228	4
SMAD9/SNRPE	-0.17743	2.124939
SMAD9/VARS	-0.16629	1.910095
SMAD9/MEX3A	-0.15663	1.732828
SMAD9/GSPT1	-0.14998	1.617983
SMAD9/CDKN2A	-0.12098	1.158015
SMAD9/TIA1	-0.10124	0.888737
EIF4E3/TIA1	-0.09948	0.866461
EIF4E3/CDKN2A	-0.08237	0.66274
EIF4E3/RPL39L	-0.06546	0.485054
SMAD9/PPARGC1B	-0.06105	0.442493
EIF4E3/VARS	-0.04791	0.324588
PPARGC1B/GSPT1	-0.04706	0.317404
SMAD9/RPL39L	-0.03013	0.185553
EIF4E3/GSPT1	-0.00869	0.047353
PPARGC1B/TIA1	0.00757	0.041006
SMAD9/EIF4E3	0.00767	0.041579
EIF4E3/PPARGC1B	0.01387	0.07795
EIF4E3/SNRPE	0.03018	0.185886
PPARGC1B/CDKN2A	0.03596	0.228633
PPARGC1B/SNRPE	0.04837	0.328549
EIF4E3/SSR1	0.0505	0.346787
PPARGC1B/SSR1	0.09558	0.817871
PPARGC1B/RPL39L	0.09751	0.841939
PPARGC1B/VARS	0.12182	1.170053
PPARGC1B/MEX3A	0.12531	1.221849
TIA1/CDKN2A	0.14612	1.551294
CDKN2A/SSR1	0.2103	2.823909
CDKN2A/GSPT1	0.22007	3.045757
RPL39L/TIA1	0.22962	3.30103
RPL39L/CDKN2A	0.2484	3.69897
MEX3A/CDKN2A	0.27066	4
MEX3A/GSPT1	0.2923	4
RPL39L/SSR1	0.30234	4
TIA1/SNRPE	0.31845	4
VARS/TIA1	0.31882	4
MEX3A/RPL39L	0.31903	4
MEX3A/SSR1	0.32379	4
VARS/CDKN2A	0.3306	4
TIA1/GSPT1	0.35332	4
RPL39L/GSPT1	0.36293	4
MEX3A/TIA1	0.36975	4
CDKN2A/SNRPE	0.38595	4
VARS/MEX3A	0.41165	4
TIA1/SSR1	0.41604	4
MEX3A/SNRPE	0.42688	4
RPL39L/SNRPE	0.45747	4
VARS/GSPT1	0.47421	4
GSPT1/SSR1	0.49599	4
VARS/RPL39L	0.49727	4
SNRPE/SSR1	0.55135	4
SNRPE/GSPT1	0.584	4
VARS/SSR1	0.62097	4
VARS/SNRPE	0.66316	4
